# YIV-906 enhances nuclear factor of activated T-cells (NFAT) activity of T cells and promotes immune checkpoint blockade antibody action and CAR T-cell activity

**DOI:** 10.3389/fphar.2022.1095186

**Published:** 2023-01-04

**Authors:** Wing Lam, Rong Hu, Shwu-Huey Liu, Peikwen Cheng, Yung-Chi Cheng

**Affiliations:** ^1^ Department of Pharmacology, Yale University School of Medicine, New Haven, CN, United States; ^2^ Yiviva Inc, New York, NY, United States

**Keywords:** YIV-906, NFAT, PD-1, PD-L1, CAR T, SHP1, SHP2

## Abstract

YIV-906 is a systems biology botanical cancer drug, inspired by a traditional Chinese herbal formulation. Results from eight Phase I/II to II clinical studies demonstrated the potential of YIV-906 to prolong survival and improve the quality of life of cancer patients. As an immunomodulator in the tumor microenvironment, YIV-906 can turn cold tumors hot and potentiate anti-tumor activity for different classes of anticancer agents; and as a cytoprotector in the GI, YIV-906 can reduce non-hematological side effects and speed up damaged tissue recovery. YIV-906 enhanced anti-PD1 action against hepatoma in mice by stimulating both innate and adaptive immunity. In a Jurkat cell-staphylococcal superantigen E (SEE)-Raji cell culture model, YIV-906 promoted T cell activation with upregulation of CD69 by enhancing NFAT activity, with or without PD1-PD-L1 interaction. YIV-906 could trigger the phosphorylation of TCR downstream signaling cascades without the involvement of TCR. YIV-906 could inhibit SHP1 and SHP2 activities, which dephosphorylates TCR downstream proteins due to the PD1-PD-L1 interaction. Therefore, YIV-906 could enhance anti-PD1 action to rescue the depressed NFAT activity of Jurkat cells due to the PD1-PD-L1 interaction. In addition, YIV-906 enhanced the NFAT activity and killing capability of Jurkat cells expressing chimeric antigen receptor (CAR-CD19^−^CD3z) toward CD19 expressing cells, such as Raji cells, with or without PD1-PD-L1 overexpression. Ingredient herb **S** (*Scutellaria baicalensis Georgi*) of YIV-906 and some **S** compounds were found to play key roles in these activities. In conclusion, YIV-906 modulates adaptive immunity by activating T effector cells mainly through its action on SHP1/2. YIV-906 could also facilitate immune checkpoint blockade therapy or CAR-T cell therapy for cancer treatment.

## Introduction

YIV-906 is a botanical drug inspired by an 1800-year-old traditional herbal medicine formulation called “Huang Qin Tang,” historically used to treat numerous gastrointestinal (GI) symptoms, including diarrhea, nausea, and vomiting. YIV-906 is composed of four medicinal plants: *Glycyrrhiza uralensis* Fisch (**G**), *Paeonia lactiflora* Pall (**P**), *Scutellaria baicalensis* Georgi (**S**), and *Ziziphus jujuba* Mill (**Z**). Eight clinical-grade preparations of YIV-906 have been manufactured using cGMP standards over the past 20 years. Batch-to-batch consistency was established and validated using quality control platforms, including Phytomics (Tilton et al., 2010) and its next-generation Mechanism-based Quality Control platform (Mech QC) that measures the consistency of bioactivities (Lam et al., 2018).

Over 250 patients with liver, pancreatic, colorectal, or rectal cancers have been treated with YIV-906 in combination with different cancer regimens (including irinotecan-based therapies, sorafenib, capecitabine, or chemo-radiation) in nine phase I/II to II clinical studies at numerous research institutions, including Yale University, Stanford University, UPMC Hillman Cancer Center, City of Hope Comprehensive Cancer Center, and Memorial Sloan Kettering (Yen et al., 2009; Saif et al., 2010; Kummar et al., 2011; Saif et al., 2014; Changou et al., 2021). A phase II randomized, double-blinded, placebo-controlled clinical trial (NCT04000737) evaluating the use of YIV-906 in combination with sorafenib as first-line therapy for hepatitis B (HBV+) patients with advanced hepatocellular carcinoma (HCC) is currently enrolling patients in the United States, Mainland China, Hong Kong, and Taiwan. YIV-906 has been observed to prolong patient survival and reduce grade 3/4 non-hematological toxicities including diarrhea, nausea, vomiting, fatigue, and hand-foot syndrome (Yen et al., 2009; Saif et al., 2010; Kummar et al., 2011; Saif et al., 2014; Changou et al., 2021).

In animal studies, YIV-906 reduced irinotecan (CPT-11)-induced intestinal inflammation by inhibiting TNFα, NF-κB, COX-2, iNOS, and IL6, while promoting intestinal stem/progenitor cell repopulation by potentiating the Wnt signaling pathway (Lam et al., 2010). In irradiation studies, YIV-906 also reduced GI toxicities and promoted GI recovery following radiation treatment (Rockwell et al., 2013). YIV-906 also selectively alters the intestinal bacterial population; however, the change in microbes does not seem to be responsible for YIV-906s action on irinotecan (Lam et al., 2014).

In addition to reducing side effects common to chemotherapy and radiation, YIV-906 can enhance the action for a broad spectrum of anti-cancer agent classes including: immune checkpoint antibodies, multi-kinase inhibitors, topoisomerase inhibitors, anti-metabolites, alkylating agents, anti-microtubule agents, and nucleoside analogs in animals (Liu and Cheng, 2012). In the presence of neoantigens caused by anti-cancer agents, YIV-906 could potentiate innate and/or adaptive immunity potentiation through multiple mechanisms of action. To enhance innate immunity, YIV-906 can potentiate interferon-gamma (IFNg) action to induce M1-like macrophage polarization simultaneously inhibiting IL4 action to induce M2 macrophage polarization (Lam et al., 2015; Yang et al., 2021). To enhance adaptive immunity, YIV-906 reduces PD1 or counteracts PD-L1 induction caused by anti-PD1, leading to higher T-cell activation-associated gene expression in the tumor (Yang et al., 2021). To help overcome immune suppression, YIV-906 reduces immune tolerance by modulating IDO activity and reducing monocytic MDSC in the tumor (Yang et al., 2021).

Here, we report that YIV-906 and its components can promote T cell activation by modulating nuclear factor of activated T-cells (NFAT) activity. Inhibition of SHP1/2 phosphatases and the induction of protein phosphorylation of T cell receptor signaling cascades could play an integral role in the mechanisms of action. Our studies also suggest that YIV-906 could enhance immune checkpoint blockade antibody action as well as CAR T cell therapy.

## Methods and materials

### Preparation of herbal extracts

YIV-906 is a complex mixture prepared using a traditional hot water extract of four medicinal plants, *Scutelleria baicalensis Georgi* (S) and *Paeonia lactiflora Pall*. (P) *Glycyrrhiza uralensis Fisch.* (G), and *Ziziphus jujuba Mill* (Z) at a ratio of 3:2:2:2, respectively. It can be consistently prepared batch-to-batch and used in clinical trials. Details of the quality control of YIV-906 can be found in our previous reports (Tilton et al., 2010; Lam et al., 2018). YIV-906 water extract (100 mg/ml) was prepared as a stock for all culture assays.

### Cell lines

RPMI1640 (Gibco, cat#11835030)+5%FBS+50ug/ml kanamycin at 37°C with 5%CO_2_ incubation was used for cell culture and experiments. Authenticated Jurkat cell lines were purchased from ATCC (Jurkat, Clone E6-1), (T lymphoblast) cells were transfected with pcDNA-PD1-Dyk DNA (Genscript Biotech Corp, OHU263220D) using Lipofectamine LTX (Thermo Fisher Scientific, Cat#15338030) and then selected by G418 200 ug/ml. Authenticated Raji cell lines were purchased from ATCC (Raji-CCL-86™), (B-lymphocyte) cells were transfected with pcDNA-PD-L1-Dyk DNA (Genscript Biotech Corp, OHU22144D) and then selected by G418 600 ug/ml. Anti-PD1-PE (BioLegend, Cat#A17188B) or Anti-PD-L1(CD274)-APC (Invitrogen, Cat#17-5983-42) were used to confirm the expression of PD1 or PD-L1 in Jurkat cells or Raji cells using a laser flow cytometer (BD, Accuri 6 plus). Jurkat-PD1 cells were transfected with NFAT luciferase reporter DNA, where four repeated NFAT response elements, GGA​GGA​AAA​ACT​GTT​TCA​TAC​AGA​AGG​CGT (SEQ ID NO. 1) oligos, were inserted into the pGL4.2 vector (Promega, Cat#E6751) and then selected with 0.1ug/ml puromycin. Jurkat-PD1 NFAT-luc TCRαβ knockout cells were generated by transfecting CRISPR/Cas9-gRNA: eSpCas9-2A-GFP (Genscript Biotech Corp, SC1818) inserted into the targeting sequence using Lipofectamine LTX (Thermo Fisher Scientific, #15338030). The targeting DNA sequences for TCRa and TCRb were CTT​CAA​GAG​CAA​CAG​TGC​TG and AGG​TCG​CTG​TGT​TTG​AGC​CA, respectively. PE anti-human α/β T Cell Receptor Antibody (BioLegend, Cat#306708) was used to confirm the knockout of TCRαβ using flow cytometry. The targeting sequences for SHP1 (PTPN6) and SHP2 (PTPN11) were ACC​TGA​TCC​CCC​ACC​CTG​C and AGG​CCT​AGT​AAA​AGT​AAC​CC, respectively. Western blot analysis was used to confirm the knockout of SHP1 and SHP2 using anti-SHP1 (BioLegend, Cat#620301) and anti-SHP2 (Cell Signaling, Cat#3397S). Jurkat CART cell establishment: The pSLCAR-CD19-CD3zeta vector (Addgene_135993), pCMV-VSV-G (Addgene_8454), and psPAX2 (Addgene_12260) were transfected into HEK293T for 48h. The lentivirus was concentrated using a Lenti-X concentrator (TakaRa, Cat#631231) according to the manual. Concentrated Lentivirus- pSLCAR-CD19-CD3zeta was transduced into Jurkat-PD1 NFAT-luc cells using polybrene 10ug/ml (Millipore, Cat#TR-1003-G) and then GFP-positive cells were randomly sorted using flow cytometry. Jurka-PD1 NFAT-luc CAR-CD19-CD3zeta clones were randomly selected when NFAT luciferase activity was increased by co-culture with Raji cells overnight.

### Stimulation for NFAT of Jurkat cells

Jurkat cells (T cells) were stably transfected with nuclear factor of activated T-cells (NFAT) luciferase reporter DNA and PD1 DNA. For stimulation, 50 ul of Jurkat cells-PD1 cells at 5 × 10^5^/ml were co-cultured with 50 ul of Raji cells at 10^6^/ml in a 1:2 ratio and staphylococcal superantigen E (**SEE**) (Toxin Technology, Cat#ET404) 1 ng/ml to 10 ng/ml, in the absence or presence of YIV-906 or its components were added to the mixed cells overnight at 37°C with 5%CO_2_ incubation. NFAT activity was determined by measuring luciferase activity chemiluminescence. 75 ul of Jurkat-PD1 cells at 10^6^ cells/well with or without wild-type Raji cells or Raji-PD-L1 cells at 2 × 10^6^ cells/well were seeded into round-bottom 96-well plates. In some experiments, InVivoSIM anti-human PD-1 (Nivolumab Biosimilar) (BioXcell, Cat#SIM0003) 18 ug/ml was added to the cells for 2 h or 24 h before drug and see treatment. 25 ul of control medium or 25 ul of 5x concentration of YIV-906 or other drugs were added to the well. 25 ul of see (5 × 10 ng/ml) was added to the wells. After overnight incubation at 37°C with 5%CO_2_, the cells were lysed using a luciferase lysis buffer which contained luciferin to generate the luminescence. The luminescence was recorded using a luminescence microplate reader. Each data point represents three experiments of triplicate samples from the NFAT luciferase reporter assay.

### Flow cytometry analysis

Jurkat-PD1 cells 2 × 10^5^/ml with or without wild-type Raji cells or Raji-PD-L1 cells (4 × 10^5^/ml) in 24-well plates were treated with YIV-906 with or without **SEE** (10 ng/ml and 30 ng/ml), for 48 h. Anti-CD69-FITC (BioLegend, Cat#310904) and anti-PD1-PE (BioLegend, Cat#A17188B) in PBS+1%BSA was used to stain the cells. Anti-PD1-PE was used to separate Jurkat-PD1 cells from mixed cells. The expression of CD69 in the FITC channel was presented as median fluorescence or total Jurkat cell population percentage using a laser flow cytometer (BD, Accuri 6 plus). Cell death of Raji cells (co-cultured with Jurkat-PD1-CAR-CD-19 cells/Raji cells at a 2:1 ratio) was determined by gating CD19^+^ Raji cells (Anti-CD19-perCP, BioLegend, #30228) and Annexin V-PE (BioLegend, #640947) positive and/or Helix-NR positive cells. Each data point represents the average mean of four experiments of triplicate samples of flow cytometry assays.

Following the treatment, the medium was collected for IL2, IFNg, and IL10 detection using fluorescence bead array (BioLegend: LEGENDplex assays, Human CD8/NK Panel, Cat#740267) according to the to the manufacturer’s instructions.

### SHP1 and SHP2 enzymatic activity

pNPP assays were used to determine the inhibitory effects of YIV-906 and its components on recombinant human SHP2(PTPN11) and SHP1(PTPN6) (LSBio, WA). Briefly, 20 ng of enzyme with different doses of YIV-906 or its components were reacted in the 100 ul reaction buffer (5 mM pNPP (p-Nitrophenyl Phosphate), 25 mM Hepes pH 7.3, 2.5 mM EDTA, 2.5 mM DTT, 100 ug/ml BSA) in a well of a 96-well plate at 37°C for 1 h. 100 ul of 2N H_2_SO_4_ were used to stop the reaction. Heated SHP1 or SHP2 (90°C for 15 min) was used as the control in parallel experiments. OD optical 450 nm was measured. The OD of wells without added drugs was normalized to 1 after subtracting the wells’ OD without enzyme. Each data point represents the average mean of three experiments of triplicate samples.

### Western blot analysis

Jurkat-PD1 cells 10^6^/ml were placed in 24-well plates and treated with YIV-906 or its constituent herbs for 45 min. The cells were collected at 1000 g centrifugation for 10 min. The cells were prepared with 2X protein loading buffer (Tris pH 6.8 1M, SDS 1%; glycerol; β-mercaptoethanol; bromophenol blue; and distilled water). The samples were then heated to 95°C for 5 min to denature the proteins prior to western blotting. SDS polyacrylamide gel electrophoresis (10% Mini-Protean TGX™ Precast Protein Gels, Bio-Rad) was used to separate the proteins according to their electrophoretic mobility. 20 ug of Protein extract per 10 µL per well was used. Migration was performed in a 1X running buffer (Tris/Glycine/SDS) at 185 V for 50 min. Proteins were transferred onto a PVDF membrane in transfer buffer (Tris-CAPS AX, methanol, SDS 10%, distilled water) at 75 V for 1 h. After blotting, the membrane was cut into two parts with an approximate size of 3 cm (height) × 9 cm (width) to fit into the blocking chamber. The upper part of the membrane was used for probing target proteins with specific antibodies (as described below), and the lower part of the membrane was used for probing GAPDH as a protein loading control for normalization. Non-specific binding sites on the PVDF membrane were blocked with a blocking solution (3% milk powder and 1X TBS-T) for 30 min. The PVDF membrane was then incubated with the primary antibody against the proteins of interest overnight at 4 °C. The primary antibodies used were as follows: P-Lck-Y394 (BioLegend, Cat#933101), P-Zap70-Y319 (Cell Signaling Technology, Cat#2717), P-LAT-Y191 (Cell Signaling Technology, Cat#3584), P-SRC(Fyn)Y416 (Cell Signaling Technology, Cat#6943), P-Pyk2-Y402 (Cell Signaling Technology, Cat#3291), and GAPDH (Cell Signaling Technology Cat# 5174, RRID:AB_10622025). The membrane was washed with TBS-T 1X and incubated with a secondary antibody with horseradish peroxidase-conjugated anti-rabbit IgG 1:5000, (Thermo Fisher Scientific Cat# A27036, RRID:AB_2536099), against the immunoglobulin corresponding to the primary antibody for 1 h at room temperature. The membrane was then washed with TBS-T 1X. The protein bands were detected using chemiluminescence (Super Signal West Dura, Thermo Scientific, Cat#PI34076) and the images were acquired using an X-ray film processor (Fuji Super RX-N). Densitometric scanning was performed using an Epson V600 scanner. ImageJ software (ImageJ, RRID:SCR_003070) was used to quantify the total intensity of the immunoreactive bands. GAPDH was used as an internal control for normalization.

### LC-MS analysis for chemical profiles of the metabolites of YIV-906

LC-MS analysis was performed using an Agilent 1200 series HPLC coupled with an AB SCIEX 4000 QTRAP mass spectrometer. The separation was conducted on an Alltima™ HP HPLC column (5 mm, 4.6 × 250 mm). The mobile phase was acetonitrile (A) and water with 0.1% formic acid (B) with gradient elution: 0 min, 5% A; 10 min, 20% A; 20 min, 25% A; 40 min, 30% A; 45 min, 35% A; 55 min, 45% A; 60 min, 70% A; 62 min, 90% A; 67 min, 90% A; 68 min, 5% A; and 75 min, 5% A. The flow rate was 1.0 ml/min, and the column temperature was set at 30°C. ESI negative mode mass spectrometry of scan rate 4000 amu/s was performed with the following ionization parameters: CAD: High; TEM:550.00°C; GS1:55.00; GS2:50.00; ihe: ON; IS: 4250.00; DP: 40.00; CES 0.00; CE: 5.00. The mass range for detection was 120–800 amu. Using a custom program integrated with MZmine software, the chemicals were identified based on their retention time, total mass, and ion pair fragment mass.

### Statistical analysis

Data was analyzed using one- or two-way analyses of variance (ANOVA) tests (GraphPad Prism 7), correlation analyses (GraphPad Prism, RRID:SCR_002798), and Student’s t-test distribution (Microsoft Excel, RRID:SCR_016137). Differences were considered statistically significant at *p* < 0.05.

## Results

### YIV-906 could modulate the nuclear factor of activated T-cells (NFAT) activity and promote CD69 expression in T cells with or without the interaction of PD1 and PD-L1

**FIGURE 1:**
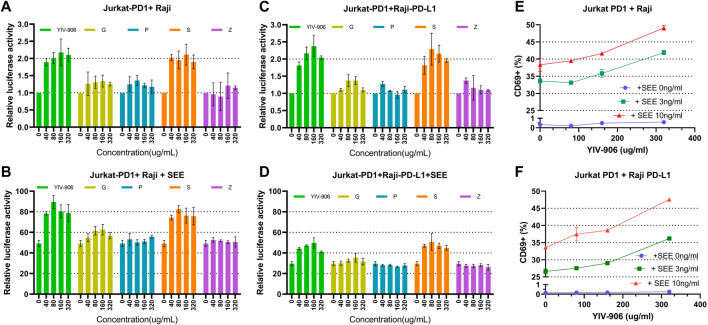
Effect of YIV-906 and its component herbs [*Glycyrrhiza uralensis* Fisch (**G**), *Paeonia lactiflora* Pall (**P**), *Scutellaria baicalensis* Georgi (**S**), and *Ziziphus jujuba* Mill (**Z**)] on NFAT mediated transcriptional activity and CD69 expression of Jurkat cells incubated with wild type Raji cells without or with staphylococcal superantigen E (**SEE**). Effects of YIV-906 and its single herb on NFAT mediated transcriptional activity of Jurkat cells incubated with Raji cells without **SEE (A)** and with **SEE (B)** were compared. The effects of YIV-906 and its single herb on NFAT mediated transcriptional activity of Jurkat-PD1 cells incubated with Raji-PD-L1 cells without **SEE (C)** and with **SEE (D)** were compared. Equivalent amounts of water extract up to 320 ug/ml were added to Jurkat cells, which were stably transfected with NFAT luciferase reporter, together with Raji cells with or without **SEE** 10 ng/ml for 24 h before luciferase activity was measured. Each data point represents the average mean of three experiments of triplicate samples from NFAT luciferase reporter assay. Effects of YIV-906 on CD69 expression of Jurkat PD1 cells, when incubated with PD-L1 overexpressed Raji cells **(E)** or Raji PD-L1 cells **(F)** in absence or presence of **SEE** antigen, are determined following 48 h incubation. The expression of CD69 was determined by flow cytometry in which FITC conjugated anti-CD69 was used to determine the expression of CD69 protein on the membrane of Jurkat cells and PE conjugated anti-PD1 was used to gate Jurkat PD1 cell from the mixture of Jurkat/Raji cells. Details of experimental procedures are given in materials and methods.

A cell culture model of Jurkat cells co-cultured with staphylococcal superantigen E (**SEE**)-Raji cells was established to examine the effects of YIV-906 on T-cell activation. In this cell culture model, YIV-906 was able to promote NFAT activity by approximately one-fold in either the absence (Figure 1A) or presence (Figure 1B) of **SEE**, which could stimulate NFAT activity about 40-fold in the range of 80 ug/ml to 320 ug/ml, which are non-toxic doses for Jurkat cells and Raji cells.

Effects of different component herbs (at equivalent YIV-906 concentrations): *Glycyrrhiza uralensis* Fisch (**G**), *Paeonia lactiflora* Pall (**P**), *Scutellaria baicalensis* Georgi (**S**), and *Ziziphus jujuba* Mill (**Z**) on NFAT-mediated transcriptional activity of Jurkat cells was compared. **S** showed a very similar dose response to YIV-906 in both the absence and presence of **SEE** (Figures 1A, B); **G** and **P** could modulate no more than 20% NFAT activity in the absence or presence of **SEE** (Figures 1A, B); and **Z** had no discernable impact on NFAT activity (Figures 1A, B). When comparing two-herb combinations or three-herb combinations (Supplementary Figures S1A–D), only the combinations that included **S** showed an approximately 1-fold enhancement of NFAT activity in the absence or presence of **SEE**. We demonstrated that YIV-906 modulates nuclear factor of activated T-cells (NFAT) activity in Jurkat cells. **S** played the most important role in the modulation of NFAT, conversely **G** and **P** might play minor roles in the modulation of NFAT.

To study the impact of PD1-PD-L1 on NFAT activity during T cell activation, Jurkat cells-PD1 cells were co-cultured with PD-L1 over-expressed Raji cells, with or without **SEE**. YIV-906, **S**, and combinations with **S** had similar effects on the basal NFAT activity of Jurkat-PD1 cells, either incubated with Raji (Figure 1A, Supplementary Figures S1A–C) or PD-L1 over-expressed Raji cells (Raji-PD-L1) (Figure 1C, Supplementary Figures S1E–G). PD1 Interactions with Jurkat cells and PD-L1 with Raji cells inhibited NFAT activity of Jurkat cells by ∼50% (Figure 1B vs. 1D). YIV-906, **S,** and combinations with **S** rescued the depressed NFAT activity of Jurkat-PD1 cells (Figure 1D, Supplementary Figures S1F, H). **G** had a slight impact on NFAT activity under the above conditions (Figures 1C,D and Supplementary Figures S2E–H).

Since NFAT is a very important T cell activation transcriptional factor, the impact of YIV-906 on T cell activation was further studied. CD69 was used as a T cell activation marker. As shown in Figure 1E, YIV-906 enhanced CD69^+^ population (Figure 1E) and the median CD69-fluorescence (Supplementary Figure S2A) of Jurkat-PD1 cells when incubated with Raji cells without **SEE**, or with 3 ng or 10 ng/ml **SEE**. The interaction between PD1 and PD-L1 reduced CD69 expression induced by **SEE**, but YIV-906 could help to restore CD69 expression in Jurkat-PD1 cells (Figure 1F and Supplementary Figure S2B). This result suggested that YIV-906 could modulate NFAT activity of T cells, thereby leading to stronger T cell activation even under PD1-PD-L1 interaction conditions. Since Jurkat cells are a leukemia T cell, their responses may not perfectly reflect the normal conditions. T cells isolated from mice spleen were used to confirm if YIV-906 and **S** could still promote T cell activation. Results demonstrated that YIV-906 and S treatment could increase CD69^+^ population of CD4^+^ cells (Supplementary Figure S2C) and CD8^+^ cells (Supplementary Figure S2D) isolated from mouse spleen. This result further supported that **YIV-906** and **S** have potential to promote T cell activation. In further, we will seek collaboration to study the impact of YIV-906 on primary human T lymphocytes.

### The action of YIV-906 and S on NFAT does not require TCRαβ

**FIGURE 2:**
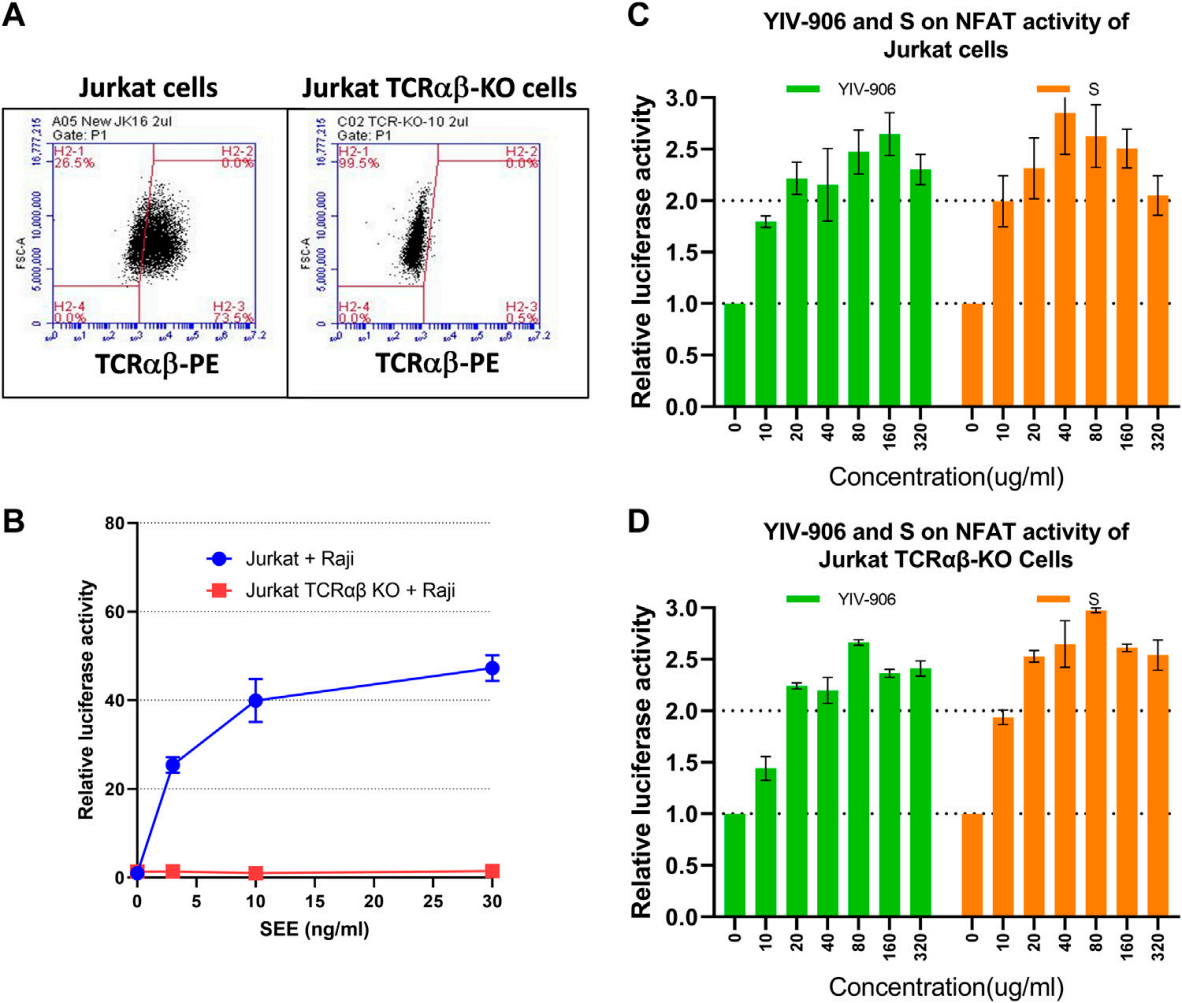
Effects of YIV-906 and **S** on NFAT mediated transcriptional activity of Jurkat cells and T cell receptor-αβ (TCRαβ) knockout Jurkat-cells. Detection of TCRαβ of Jurkat and Jurkat TCRαβ knockout cells **(A)** by using FITC conjugated anti-TCRαβ and flowcytometry is presented. Effect of **SEE** on NFAT activity of Jurkat cells or Jurkat TCRαβ KO cells together with Raji wild-type cells **(B)**. Effects of YIV-906 and **S** on NFAT-mediated transcriptional activity of Jurkat-PD1 cells **(C)** and Jurkat-PD1 TCRαβ knock-out cells **(D)** are also shown. Each data point represents the average mean of three experiments of triplicate samples from NFAT luciferase reporter assay. Details of experimental procedures are given in materials and methods.

To investigate whether YIV-906 and its components activates NFAT activity through direct activation of TCR, the TCRαβ gene was knocked out from Jurkat-PD1 cells using CRISPR/Cas9 technology (Figure 2A). Jurkat-PD1 TCRαβ knockout (KO) cells did not respond to **SEE** when co-cultured with Raji cells (Figure 2B). As shown in Figures 2C, D, YIV-906 and S had very similar impacts on NFAT activity in Jurkat-PD1 and Jurkat-PD1 TCRαβ KO cells. These results suggest that TCRαβ are not essential for the action of YIV-906 and its components on NFAT. YIV-906 and its components may have direct downstream targets of TCR αβ.

10R-VIVIT peptide, which is a cell-permeable peptide, could inhibit NFAT activity by blocking calcineurin-mediated dephosphorylation of NFAT. 10R-VIVIT at 25 uM, a non-toxic dose, could inhibit about 70% of NFAT activity of Jurkat-PD1 cells when co-cultured with Raji or Raji-PD-L1 cells (Supplementary Figure S3A). Under 10R-VIVIT (25uM) treatment conditions, YIV-906 and **S** could still promote NFAT activity of Jurkat-PD1 cells with similar magnitude (about 2 folds) without 10R-VIVIT treatment conditions (Supplementary Figure S3B, 3C). Therefore, calcineurin might not be the major target for YIV-906 and **S**.

### YIV-906 and its component herbs could induce protein phosphorylation of T cell receptor downstream cascades

**FIGURE 3:**
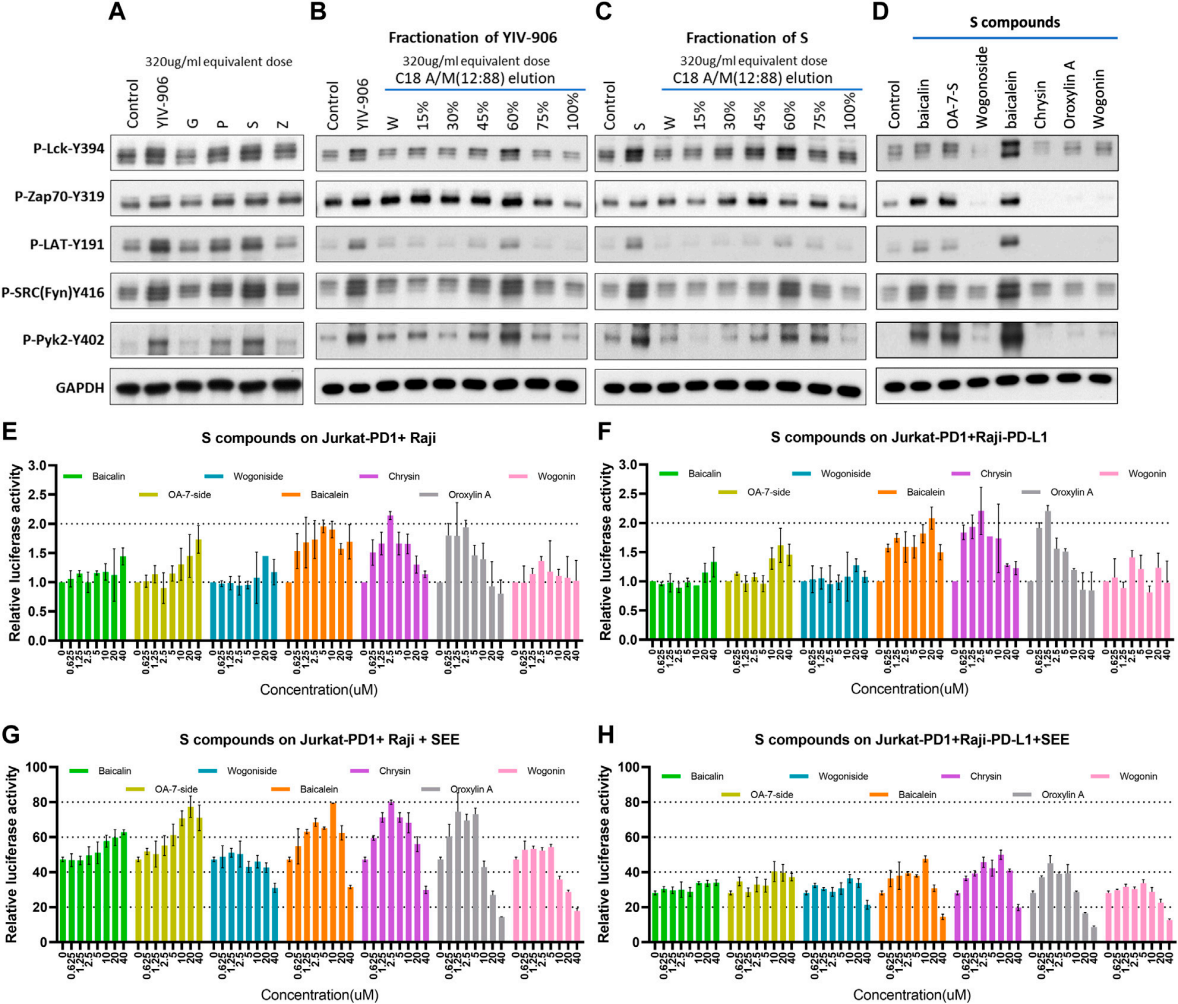
Effect of YIV-906 and its component on protein phosphorylation of T cell receptor signaling cascades and NFAT mediated transcriptional response. Effect of YIV-906 and its component herbs **(A)**, the fractions of YIV-906 **(B)**, **S (C)**, and **S** compounds **(D)** on protein phosphorylation of T cell receptor signaling cascades including, Lck, Fyn, Zap70, LAT, and Pyk2. Jurkat cells were treated with YIV-906 and its component herbs, the fractions of YIV-906 and **S** at an equivalent dose of 320ug/ml for 45min. C18 solid phase extract column was used to fractionize YIV-906 and **S** by eluting with water and increasing percentage of acetonitrile/methanol (A/M). All eluted fractions were dried and reconstituted with water to an equivalent concentration at 100 mg/ml. 20 uM of **S** was used to treat Jurkat cells for 45 min. Western blot analysis was used to determine the protein phosphorylation using specific antibodies. GAPDH was used to normalize the protein loading. Cropped blots are used in this figure and they have been run under the same experimental conditions. Effects of S compounds on NFAT mediated transcriptional activity of PD1 overexpressed Jurkat cells incubated with Raji cells **(E,G)** and PD-L1 overexpressed Raji cells **(F,H)** without and with **SEE**. S compounds, up to 40 uM, were added to Jurkat-PD1 cells, which were stably transfected with NFAT luciferase reporter and co-culturing with Raji cells or Raji-PD-L1 cells with **SEE** 10 ng/ml for 24 h before luciferase activity was measured. Details of experimental procedures are given in materials and methods.

To test whether YIV-906 and its components could directly impact downstream signaling of TCR, their effects on the phosphorylation of T cell receptor downstream cascades, including Lck, Zap70, LAT, Fyn, and Pyk2 in Jurkat cells was examined using western blot analysis. As shown in Figure 3A, treatment with YIV-906 at a dose of 320 ug/ml for 45 min induced the phosphorylation of Lck, Zap70, LAT, Fyn, and Pyk2. By comparing individual herb effects at equivalent concentrations (320 ug/ml), component **S** was found to play a major role in protein phosphorylation induction by YIV-906 (Figure 3A). Using a C18 column to fractionate YIV-906, higher relative induction activity of protein phosphorylation was observed in 45%–75% acetonitrile/methanol (A/M) fractions (Figure 3B). **S** was found to have an activity profile similar to YIV-906 (Figure 3C). The 45% A/M fraction of **S** had a strong effect on P-Zap70-Y319, whereas the 60% A/M fraction of **S** had a strong effect on P-Lck-Y39, P-LAT-Y191, P-SRC(Fyn)-Y416, and P-Pyk2-Y402 (Figure 3C). The chemicals present in the **S** fractions are listed in Supplementary Figure S4A and they had been reported in our previous reports (Ye et al., 2007; Zhang et al., 2010). In Supplementary Figure S4A, protein phosphorylation induced by these fractions was indicated using red upward arrows. It should be noted that these chemicals might have different modes of action on the targets.

Some chemicals from active **S** fractions were studied for their ability to induce protein phosphorylation of T cell receptor downstream cascades, including Lck, Zap70, LAT, Fyn, and Pyk2 in Jurkat cells using western blot analysis. Baicalein strongly induced phosphorylation of all the examined proteins (Figure 3D). Both baicalin and oroxylin A 7-O-beta-D-glucuronide (OA-7-S), at 40 uM, showed some induction effects on P-Zap70-Y394, P-SRC(Fyn)-Y416, and P-Pyk2-Y402 (Figure 3D). These compounds potentially could play a critical role in T cell activation by YIV-906. Some **S** compounds promoted NFAT activity: Oroxylin A 7-O-beta-D-glucuronide (OA-7-side), baicalein, oroxylin A, and chrysin were able to modulate the basal NFAT activity of Jurkat-PD1 cells incubated with Raji, or PD-L1 over-expressed Raji cells (Figures 3E, F). The measured modulatory effects were independent of TCRαβ expression (Supplementary Figure S4B, C), but the optimum doses were different. Oroxylin A 7-O-beta-D-glucuronide at 40 uM promote NFAT activity by 2 folds, whereas baicalein, oroxylin A, and chrysin required only 10 to 2.5 uM to have a similar impact on NFAT. In the presence of **SEE**, oroxylin A 7-O-beta-D-glucuronide, baicalein, oroxylin A, and chrysin showed similar promotional effects as they did on basal NFAT activity. It was also observed that wogonoside, baicalein, oroxylin A, and chrysin inhibited **SEE**-triggered NFAT activity at concentrations from 20 to 40 uM (Figures 3G, H). Some compounds mentioned above exhibited a biphasic effect on NFAT activity, possibly because these compounds might have multiple modes of action on their target(s) that are associated with the NFAT pathway. In the tumor microenvironment, overall activity might depend on the composition and internal interactions of compounds within YIV-906, hence no single compound or target can simply explain all activities.

### YIV-906 could modulate SHP1 and SHP2 activities, and the response of NFAT to YIV-906 might be dependent on SHP1/2

**FIGURE 4:**
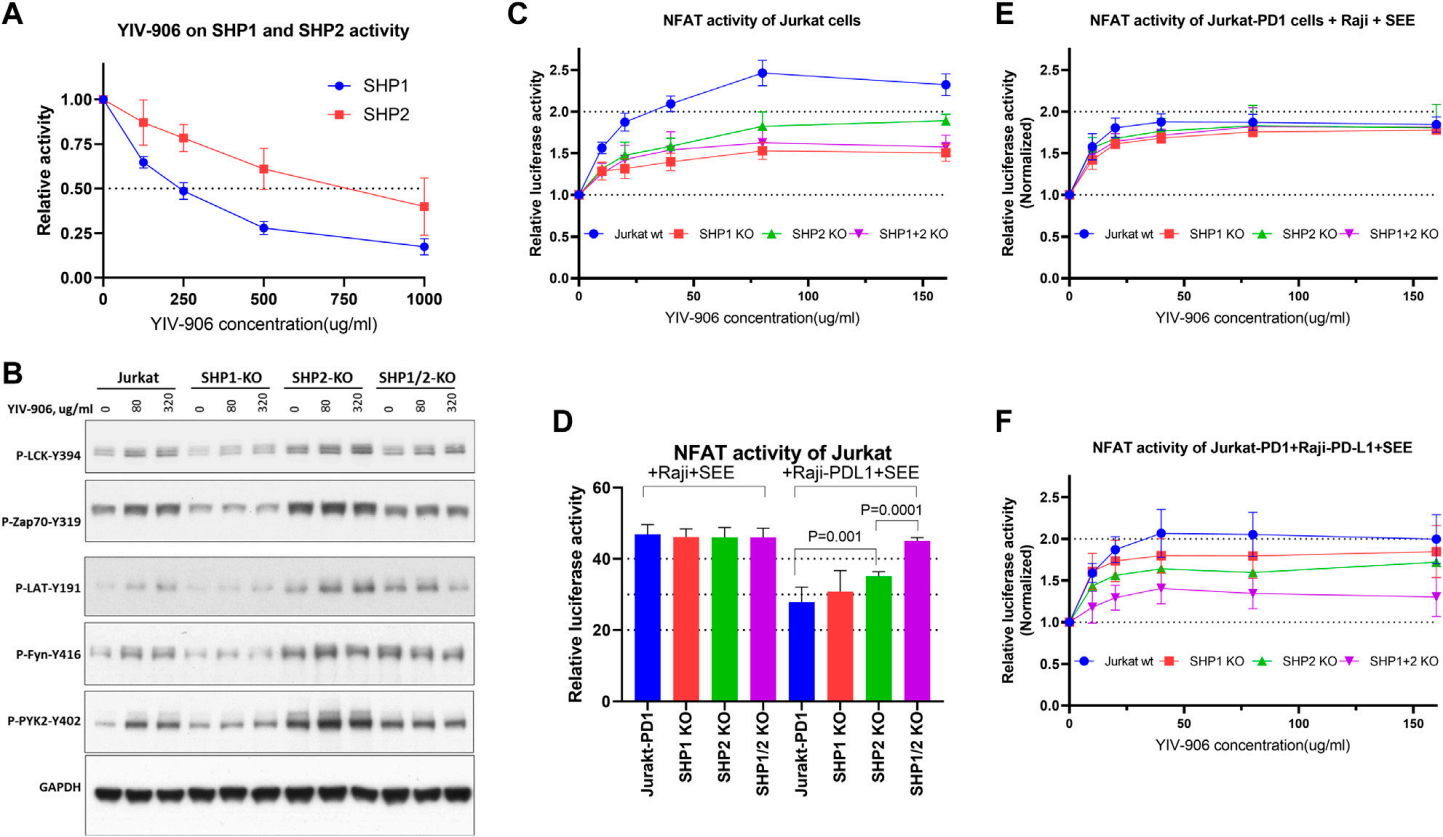
Effect of YIV-906 on TCR downstream protein phosphorylation and NFAT mediated transcriptional activity of Jurkat cells in the presence or absence of SHP1 and/or SHP2. **(A)** Effect of YIV-906 on the enzymatic activities of SHP1 and SHP2. Activities of SHP2 and SHP2 were determined by the p-Nitrophenyl Phosphate (pNPP) colorimetric assay. Each data point represents the mean of triplicate samples. **(B)** Effect of YIV-906 on protein phosphorylation of T cell receptor signaling cascades including, Lck, Fyn, Zap70, LAT, and Pyk2. Jurkat cells were treated with YIV-906 for 45 min. Western blot analysis was used to determine the protein phosphorylation using specific antibodies. GAPDH was used to normalize the protein loading. **(C)** NFAT response of Jurkat-PD1 cells with SHP1 and/or SHP2 knockout to YIV-906. **(D)** NFAT activity of Jurkat-PD1 cells with SHP1 and/or SHP2 knocked-out co-cultured with Raji wt or Raji-PD-L1 plus **SEE**. **(E)** NFAT response of Jurkat-PD1 cells with SHP1 and/or SHP2 knocked-out co-cultured with Raji wt plus **SEE**. **(F)** NFAT response of Jurkat-PD1 cells with SHP1 and/or SHP2 knocked-out co-cultured with Raji-PD-L1 wt plus **SEE** 10 ng/ml. Each data point represents the average mean of four experiments of triplicate samples from NFAT luciferase reporter assay. Details of experimental procedures are given in materials and methods.

SHP2 (SH2-containing phosphatase 2, a protein tyrosine phosphatase) is an immediate downstream mediator of PD1 (Marasco et al., 2020; Patsoukis et al., 2020). Once PD1 binds to PD-L1, PD-1 will phosphorylate its immune receptor tyrosine–based inhibitory motif (ITIM) and immune receptor tyrosine–based switch motif (ITSM) to recruit and activate SHP2 (Marasco et al., 2020; Patsoukis et al., 2020). Activated SHP2 initiates T-cell inactivation by dephosphorylating TCR downstream cascade proteins (Marasco et al., 2020; Patsoukis et al., 2020). In the absence of SHP2, SHP1 can replace SHP2 (Celis-Gutierrez et al., 2019). SHP2 inhibitors could block PD1 action and promote T-cell activation for facilitating cancer treatment (Zhao et al., 2019; Yuan et al., 2020). As shown in Figure 4A, YIV-906 showed inhibitory effects on both SHP1 and SHP2 enzyme activities, with stronger potency against SHP1. **G** and **S** component herbs of YIV-906 were found to be the key herbs responsible for SHP1 and SHP2 inhibition (Supplementary Figure S5A, B); and some individual chemical compounds in **G** and **S** were found to have inhibitory effects on SHP1 and SHP2 (Supplementary Figure S5C, D). These compounds demonstrate selectivity for SHP1 and SHP2, for example, isoliquiritigenin was more potent against SHP1 than SHP2, whereas glycyrrhizic acid showed stronger inhibition of SHP2 than SHP1 (Supplementary Figure S5C, D). Baicalin, oroxylin A 7-O-beta-D-glucuronide, wogonoside, baicalein, chrysin, and oroxylin A from **S** showed stronger inhibitory effects on SHP2 than on SHP1 (Supplementary Figure S5C, D).

To determine whether TCR downstream protein phosphorylation induced by YIV-906 is dependent on the presence of SHP1 and/or SHP2, SHP1 and/or SHP2 genes were knocked out in Jurkat-PD1 cells using CRISPR-gRNA (Supplementary Figure S6). It appeared that SHP2 and SHP1/2 knockout cells had higher basal levels of protein phosphorylation (Figure 4B and Supplementary Figures S7A–G). SHP2 might be a critical enzyme for maintaining lower levels of TCR downstream protein phosphorylation as well. Comparing to Jurkat cells, SHP1 knockout reduced the phosphorylation of all proteins triggered by YIV-906 (Figure 4B and Supplementary Figures S7A–G). SHP2 knockout did not affect the protein phosphorylation of LCK and LAT triggered by YIV-906 (Figure 4B and Supplementary Figures S7A–G). However, the level of phosphorylation of Zap70, Fyn, and PYK2 in SHP2 knockout cells only slightly increased under YIV-906 treatment (Figure 4B and Supplementary Figures S7A–G). It is interesting to note that LCK, Zap70, and PYK2 in SHP1/2 knockout cells had little or no response to YIV-906 and that a high dose of YIV-906 decreased LAT and Fyn phosphorylation (Figure 4B and Supplementary Figures S7C–G). Overall SHP1 and SHP2 could have different impacts on regulating the basal levels of protein phosphorylation of TCR downstream. Both SHP1 and SHP2 were important to YIV-906 triggering Zap70 and Fyn phosphorylation (Supplementary Figure S7G). Additionally, SHP1 and SHP2 double knockout minimized TCR downstream protein phosphorylation triggered by YIV-906 (Supplementary Figure S7G).

YIV-906’s impacts on promoting NFAT response by SHP1 and SHP2 were studied. As shown in Figure 4C, knockout of SHP1 and/or SHP2 in Jurkat cells reduced but did not completely block NFAT activity triggered by YIV-906 in the absence of **SEE**. Under PD1-PD-L1 interaction conditions, single knockout of SHP2 but not SHP1 significantly increased NFAT activity (Figure 4D). SHP1/2 knockouts completely rescued the depressed NFAT activity due to PD1-PD-L1 interaction (Figure 4D). These results support previous findings that PD-1 preferentially binds with SHP-2 over SHP-1 to inhibit TCR response (Celis-Gutierrez et al., 2019). Under Jurkat-PD1-Raji plus **SEE** conditions, knocking-out SHP1 and/or SHP2 did not have a significant impact on the cells’ NFAT response to YIV-906 (Figure 4E). When Jurkat-PD1 cells were co-cultured with Raji-PD-L1 plus **SEE**, SHP1 or SHP2 knockout only slightly decreased the NFAT response to YIV-906 (Figure 4F), but SHP1/2 double knockout significantly reduced the NFAT response to YIV-906. Overall, our results indicated that YIV-906 could overcome PD1-PD-L1 suppression and promote NFAT activity by affecting SHP1 and SHP2 activity.

### YIV-906 could further enhance anti-PD1 antibody action to promote NFAT activity in T cells

**FIGURE 5:**
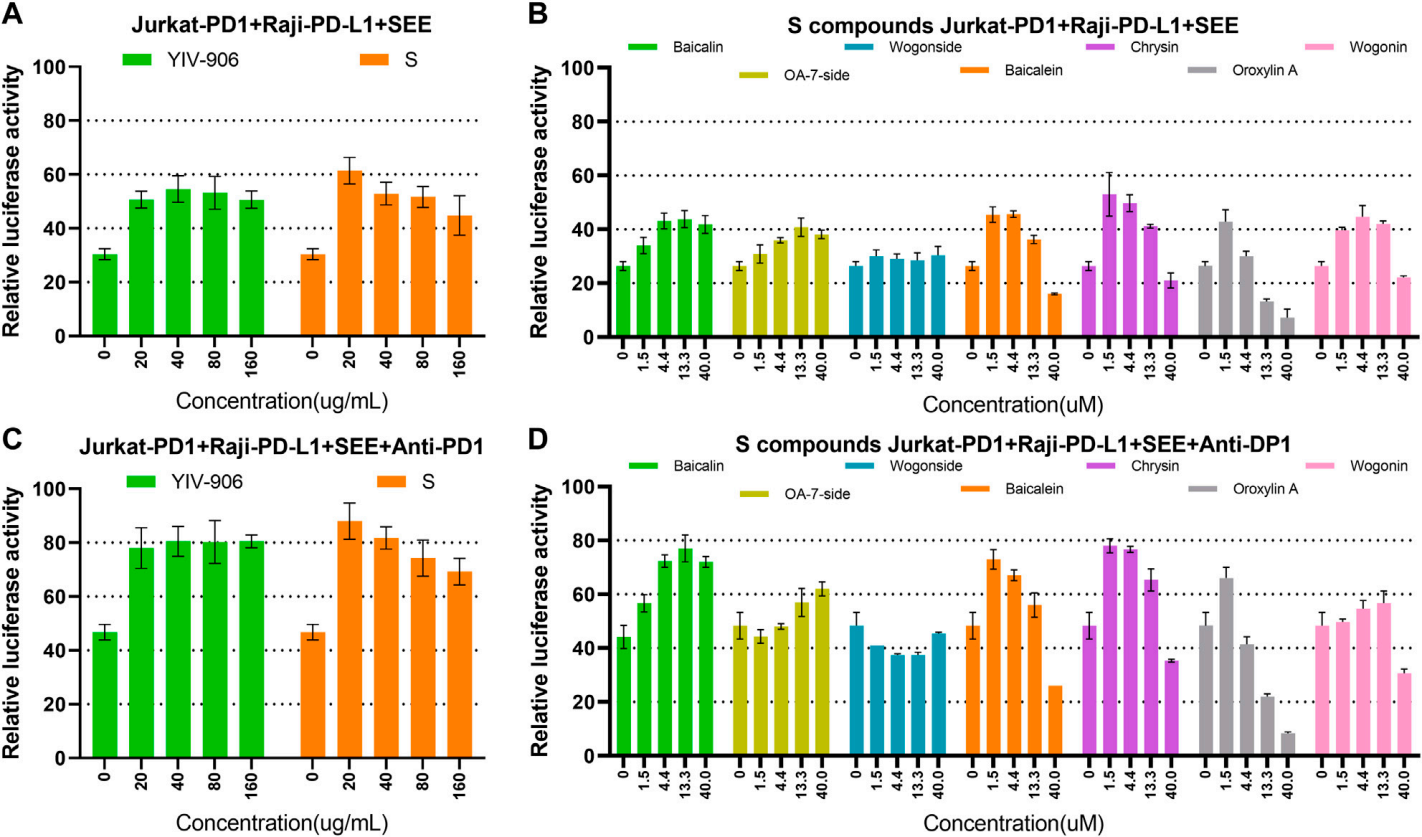
Effects of YIV-906, S, and S compounds with or without anti-PD1 on NFAT mediated transcriptional activity of Jurkat cells. **(A–B)** Effects of YIV-906 **(A)**, **S (A)** and S compounds **(B)** on NFAT mediated transcriptional activity of Jurkat-PD1 cells incubated with Raji-PD-L1 cells and **SEE** 10 ng/ml. **(C–D)** Effects of YIV-906 **(C)**, **S (C)** and S compounds **(D)** on NFAT mediated transcriptional activity of Jurkat-PD1 cells incubated with Raji-PD-L1 cells, **SEE** 10 ng/ml and anti-PD1 18 ug/ml. Cells and drugs were incubated for 24 h before luciferase activity was measured. Each data point represents the average mean of three experiments of triplicate samples from NFAT luciferase reporter assay. Details of experimental procedures are given in materials and methods.

As YIV-906 can modulate the antitumor activity of anti-PD1 against tumors in animal models, we investigated whether YIV-906 could cooperate with anti-PD1 to promote NFAT activity for T cell activation. When co-cultured with Jurkat-PD1 and Raji plus **SEE**, the addition of anti-PD1 (non-therapeutic biosimilar antibody to nivolumab , with the same variable regions ) did not affect the NFAT response of Jurkat-PD1 cells to YIV-906, **S,** or **S** compounds (Supplementary Figures S8A–D)*.* As shown earlier, co-cultured Jurkat-PD1 cells and Raji-PD-L1 cells inhibited the NFAT activity of Jurkat cells triggered by **SEE** (Figures 5A, B). YIV-906, **S**, and **S** constituent compounds restored any depressed NFAT activity in Jurkat-PD1 cells (Figures 5A, B). As expected, anti-PD1 rescued the depressed NFAT activity of Jurkat-PD1 cells due to PD1 and PD-L1 interactions (Figures 5C, D). Most importantly, YIV-906, **S**, and **S** compounds (with different optimum dose) could further enhance anti-PD1 action to promote NFAT activity in Jurkat PD1 cells (Figures 5C, D). The above results demonstrated that YIV-906 and its components could cooperate with anti-PD1 to promote T-cell activation for immune therapy.

### YIV-906 could modulate chimeric antigen receptors triggered NFAT activity of T cells

It is well known that MHC-antigen-TCR interaction recruits CD3 receptors to transduce signals for NFAT activation. Most chimeric antigen receptors (CAR) are composed of an antigen recognition domain, an extracellular hinge region, a transmembrane domain, and co-stimulatory domains such as immunoreceptor tyrosine–based activation motifs (ITAM) of CD3ζ(zeta) (Lindner et al., 2020). Once a chimeric antigen receptor (CAR) binds to its target, a signal is transduced to its ITAM of CD3ζ which further stimulates the downstream cascade leading to NFAT activation (Lindner et al., 2020). Because MHC-antigen-TCR interactions or CAR-target interactions both have the same downstream cascade stimulating NFAT activity, and YIV-906 demonstrated the potential to enhance MHC-antigen-TCR triggered NFAT response, we asked whether YIV-906 could also modulate CAR-triggered NFAT activity. To study this, Jurkat-PD1 NFAT luciferase receptor cells were transduced with CAR-CD19^−^CD3ζ(zeta) lentivirus to target CD19 on Raji cells.

**FIGURE 6:**
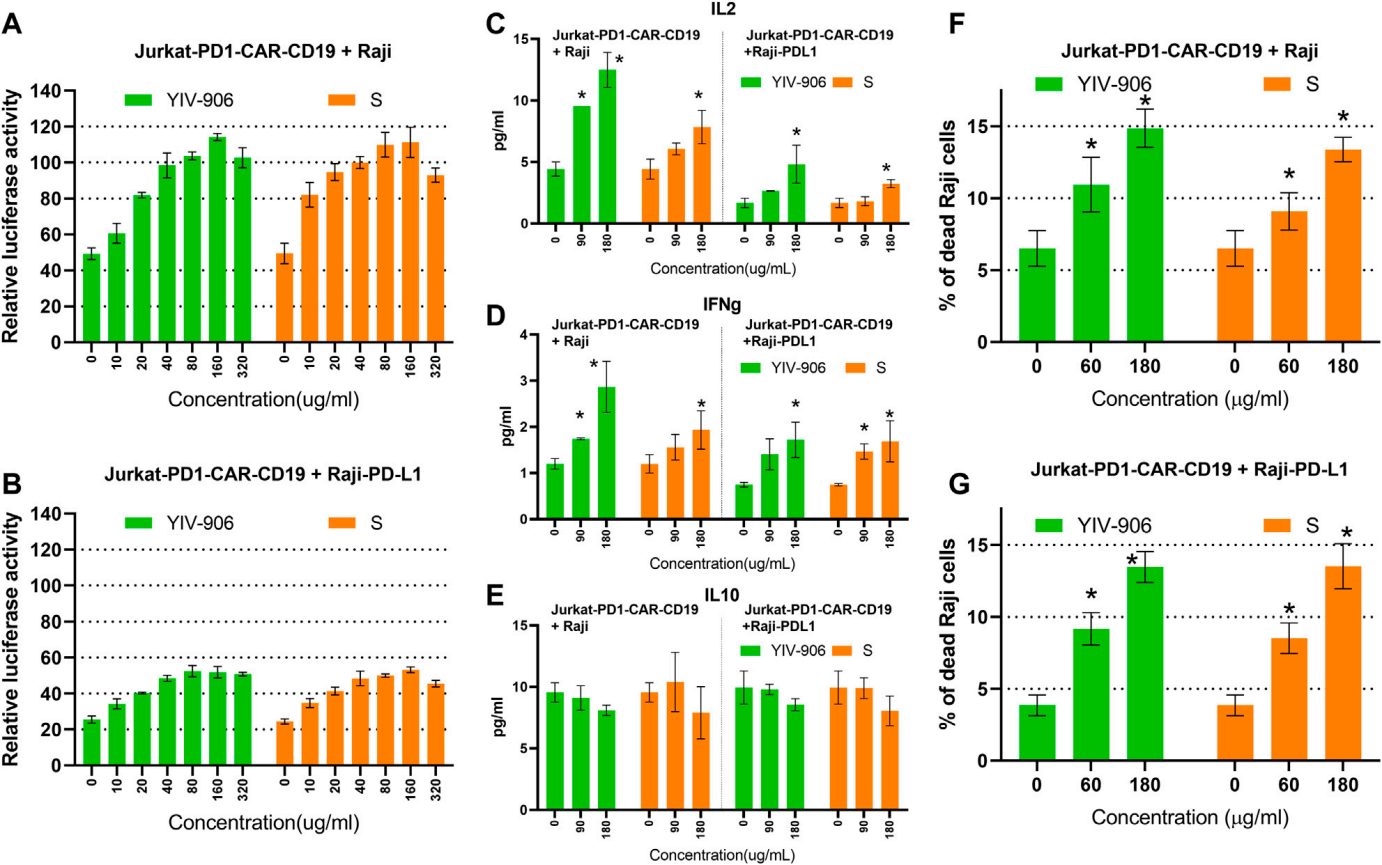
Effects of YIV-906, S, and S compounds on NFAT mediated transcriptional activity and of Jurkat cells and cell death of Raji cells triggered by co-culturing Jurkat cells over-expressing chimeric antigen receptor (CAR-CD19^−^CD3z) and Raji or Raji-PD-L1 cells. Effects of YIV-906 and **S** on NFAT-mediated transcriptional activity of Jurkat-PD1-CAR-CD19 cells incubating with Raji cells **(A)** or Raji-PD-L1 cells **(B)**. Effect of YIV-906 and **S** on IL2 **(C)**, IFNg **(D)** and IL10 **(E)** production when Jurkat-PD1-CAR-CD19 cells were co-cultured with Raji or Raji PD-L1. Effects of YIV-906 and **S** on cell death triggered by incubating Jurkat-PD1-CAR-CD19 cells with Raji cells **(F)** or Raji-PD-L1 cells **(G)**. Each data point represents the average mean of triplicate samples from NFAT luciferase reporter assay or flow cytometry assay. YIV-906, S (at concentrations up to 320 ug/ml water extract) were added to Jurkat-PD1-CAR-CD19 cells, which were stably transfected with NFAT luciferase reporter, together with wild type Raji cells or Raji-PD-L1 cells for 24 h before luciferase activity or cytokines of medium were measured. Cytokine fluorescence bead assays were sued to quantify IL2, IFNg, and IL10 of medium following treatments. Annexin V-PE and helix-NR-APC were used to stained dead cells of CD19^+^ Raji cells using flow cytometry and result labeled with * when *p* values of T-test was less than 0.05. Details of experimental procedures are given in materials and methods.

YIV-906, **S**, and **S** compounds had similar effects on promoting the basal activity of NFAT in both Jurkat-PD1-CAR-CD19 cells (Supplementary Figures S9A, B) and Jurkat-PD1 cells alone (Figure 2C and Supplementary Figures S4B). When Jurkat-PD1-CAR-CD19 cells were co-cultured with Raji cells, a 40-fold increase in NFAT-driven luciferase activity was induced (Figure 6A). Under these conditions, YIV-906, **S**, and **S** compounds further promoted NFAT activity triggered (Figure 6A and Supplementary Figures S9C). PD1 and PD-L1 interactions depressed NFAT activity triggered by the interaction CAR-CD19 and CD19 (Figure 6B). YIV-906, S, and S constituent compounds (oroxylin A 7-O-beta-D-glucuronide, wogonoside, baicalein, oroxylin A, and chrysin) could restore this depressed NFAT activity (Supplementary Figures S9D). Depending on the compound, the optimum dose for modulation may be different.

The production of IL2, IFNg and IL10 were used to monitor the response of Jurkat-PD1-CAR-CD19 cells with or without co-culturing Raji cells or Raji PD-L1 cells. As predicted, co-culturing Jurkat-PD1-CAR-CD19 cells with Raji cells could trigger higher production of IL2 and IFNg as comparing to Jurkat-PD1 plus Raji cells or Raji PD-L1 cells as well as their single culture (Figures 6C, D, Supplementary Figures S10A–F). YIV-906 and **S** could further enhance production of IL2 and IFNg production when

Jurkat-PD1-CAR-CD19 cells were co-cultured with Raji cells (Figures 6C, D). When Jurkat-PD1-CAR-CD19 cells were co-cultured with Raji PD-L1 cells, PD1 and PD-L1 interaction depressed IL2 and IFNg production (Figures 6C, D). YIV-906 and **S** could also promote IL2 and IFNg production under PD1 and PD-L1 interaction conditions (Figures 6C, D). YIV-906 and **S** had no big impacts on IL10 production in all culturing conditions (Figure 6E and Supplementary Figures S10G–I). Our results indicated that YIV-906 and **S** could further promote CAR T cell activation by enhancing IL2 and IFNg production when CAR T cells interact to its target cells.

We further demonstrated that YIV-906 could promote Jurkat-PD1-CAR-CD19 cells to kill Raji cells. As shown in Supplementary Figures S11A, B, Raji or Raji-PD-L1 cell death could be increased by increasing Jurkat-PD1-CAR-CD19 cells in the co-culture. It should be noted that PD1-PD-L1 interaction did reduce Raji-PD-L1 cell death triggered by Jurkat-PD1-CAR-CD19 cells (Fig S11A and S11B). YIV-906 could enhance the Jurkat-PD1-CAR-CD19 cells’ killing capability on either Raji or Raji-PD-L1 cells (Figures 6F, G and Supplementary Figures S11A, B). Among the component herbs, **S** was found to play a key role of YIV-906 to promote Raji or Raji-PD-L1 cell death (Figures 6F, G and Supplementary Figures S11C, D) caused by Jurkat-PD1-CAR-CD19 cells.”

K562 cells, which are myelogenous leukemia cell, did not express CD19 as comparing to Raji cells (Supplementary Figures S11E). When K562 cells were co-cultured with Jurkat-PD1-CAR-CD19 cells, no induction of NFAT activity or K562 cell death was observed (Supplementary Figures S11F, G). In addition, YIV-906 and **S** did not promote cell death of K562 cells when co-cultured with Jurkat-PD1-CAR-CD19 cells. This result demonstrated that Jurkat-PD1-CAR-CD19 cells had selective cytotoxicity on cells with CD19 expression.

Baicalein, chrysin, and oroxylin A of S (up to 10uM, did not cause significant cell death in Raji (Supplementary Figures S12A) or Raji-PD-L1 (Supplementary Figures S12B) when co-cultured, however these compounds promoted the cell killing capability of Jurkat-PD1-CAR-CD19 cells on Raji (Supplementary Figures S12C) or Raji-PD-L1 (Supplementary Figures S12D). These results suggest that YIV-906 and its components might have potential to facilitate CAR T cell therapy for cancer treatment.

## Discussion

In recent years, immunotherapy has led to many breakthroughs in cancer treatment. Many therapeutic antibodies and CAR T-cell therapies have been approved for treating different cancer types. Pembrolizumab and nivolumab are the only approved immunotherapies for pancreatic cancer and colon cancer subtypes, where criteria must have metastatic microsatellite instability-high (MSI-H) or mismatch repair deficiency (dMMR) (Overman et al., 2017; Andre et al., 2020). In general, effector T-cell activation is the key determinant of the cancer immunotherapies’ success. However patient responses to immunotherapy are not equal and certain tumor types can have lower responses rates to immune checkpoint inhibitors as compared to others. For example patients with tumors with high PD-L1 expression may have a higher response rate to anti-PD1 antibodies than those with lower expression of PD-L1 (Patel and Kurzrock, 2015). Therefore, many new targets for immunotherapy are currently in clinical trials to determine the optimal treatment. Currently hundreds of regimens are being tested to determine whether they can further promote effector T-cell activation and improve immunotherapy efficacy.

Eight clinical trials using YIV-906 as an adjuvant have been completed with encouraging results (Yen et al., 2009; Saif et al., 2010; Kummar et al., 2011; Saif et al., 2014; Changou et al., 2021). In animal studies, we have demonstrated that YIV-906 can turn “cold tumors” into “hot tumors” when combined with chemotherapy or immune checkpoint antibodies such as anti-PD1 and anti-PD-L1. Both innate and adaptive immune responses to tumors can be enhanced by YIV-906 during combination treatment. In the case of innate immunity, YIV-906 and its components potentiate IFNg action to polarize macrophages into becoming M1-like macrophages, while also inhibit IL4 action which prevents M2-like macrophage polarization (Lam et al., 2015; Yang et al., 2021).

In this study, we demonstrated that YIV-906 and its components modulates NFAT activity for T-cell activation. Since YIV-906 still induces NFAT activity in TCRα/β KO Jurkat cells, the critical factor(s) stimulated by YIV-906 should be downstream of TCR. By detecting the phosphorylation of TCR downstream proteins, we found that YIV-906 and compounds in **S** played key roles in inducing the phosphorylation of TCR downstream proteins, including LCK, Zap70, LAT, FYN, and PYK2. We previously reported YIV-906 and its components inhibit phosphatase(s) and prolong ERK1/2 phosphorylation to enhance the action of sorafenib (Lam et al., 2015). Using *in silico* analysis, YIV-906 metabolites were suggested to have a high potential for hitting DUSP3, DUSP5, and DUSP7 phosphatases (Liu et al., 2019). Many inhibitory receptors, including PD1, recruit SHP1 and/or SHP2 phosphatases *via* the phosphotyrosine-based immunoreceptor tyrosine-based inhibitory motif (ITIM) and immunoreceptor tyrosine-based switch motif (ITSM) to inhibit TCR downstream signaling (Chemnitz et al., 2004). SHP1 and SHP2 can limit T cell activation through dephosphorylation of downstream cascade proteins (Lorenz, 2009). We investigated whether YIV-906 promoted T cell activation through SHP1/2 phosphatase(s) inhibition. Our results showed that YIV-906 and certain compounds modulates the enzymatic activity of both SHP1 and SHP2. Using SHP1 and/or SHP2 KO Jurkat cells, we further demonstrated that the induction of TCR protein phosphorylation triggered by YIV-906 was partially dependent on SHP1 and/or SHP2 under PD1-PD-L1 suppressed condition. It was also demonstrated that SHP1 and SHP2 have different effects on the phosphorylation of different TCR downstream proteins. In the absence of **SEE**, SHP1 and/or SHP2 KO cells showed a lower NFAT response to YIV-906. In addition, SHP1/2 double knockout cells had much lower responses to YIV-906 than SHP1 or SHP2 single knockout cells under the PD1-PD-L1 interaction condition. These results further support the hypothesis that SHP1/2 could be one of the YIV-906s key targets responsible for the induction of TCR downstream protein phosphorylation and NFAT modulation. Since SHP1 and SHP2 could regulate NFAT signaling and they express in many cell types, we are planning to investigate if YIV-906 could also affect NFAT signaling in other cells type, such as NFAT5 activity on Raji cells. It should be noted that LCK phosphorylation and NFAT of SHP1/2 KO cells still showed some response to YIV-906, which suggests that SHP1 and SHP2 were not the only factors responsible for the NFAT modulation caused by YIV-906. Perhaps low-level LCK phosphorylation could still transduce some artifact signal to NFAT. Further investigation is needed to identify the additional mechanisms of action.

SHP2 is the predominant downstream modulator of PD1 to execute immune suppression when PD1 interacts with PD-L1 (Marasco et al., 2020; Patsoukis et al., 2020), SHP2 inhibitors are believed to have the ability to reduce immune resistance due to PD1-PD-L1 interactions. Currently, several clinical trials have been initiated to test SHP2 inhibitors, such as BBP-398 plus nivolumab (NCT05375084), RMC-4630 (SHP2-inhibitor) plus LY3214996 (ERK-inhibitor) (NCT04916236), ET0038 (NCT04528836 and NCT05354843), JAB-3068 (NCT03565003), and ERAS-601 (NCT04670679) as monotherapy or combination treatments for different types of cancer. However, SHP1 could replace SHP2 to transduce the PD1 signal for suppressing TCR downstream protein phosphorylation when SHP2 is knocked out (Celis-Gutierrez et al., 2019). In addition, SHP1 is the predominant executor of the B and T lymphocyte attenuator (BTLA), which interacts with the herpes virus entry mediator (HVEM or TNFRSF14) of APC cells (Steinberg et al., 2011). Activation of SHP1 can also depress TCR signaling (Lorenz, 2009). Furthermore, SHP1 is a key inhibitor of SIRPα (on macrophage)-CD47 (on tumor) for “don’t eat me” signaling (Myers et al., 2020). SHP1 loss increased macrophage phagocytosis of tumor cells, the ratio of effector to regulatory T cells in the E0771 tumor model, and IFNg in the MC38 tumor model (Myers et al., 2020).

Because of its dual activity on SHP1 and SHP2, YIV-906 offers an appealing alternative to single-action SHP1 or SHP2 specific inhibitors to address the multiple types of immune resistance existing in a complex tumor microenvironment. Furthermore, YIV-906 could also modulate other immune suppression pathways, such as IDO, which activates MDSC (Yang et al., 2021).

Previously we demonstrated that YIV-906 could strongly enhance anti-PD1 antibody action against Hepa 1-6 tumors (mouse hepatoma) in mice. The combination of YIV-906 and anti-PD1 eradicated Hepa 1-6 tumors in mice, and no tumor re-growth was observed when mice were re-challenged with Hepa 1-6 cells, but not when challenged with a second tumor type, such as CMT167 or Pan02 cells (Yang et al., 2021). This suggests that the combination of YIV-906 and anti-PD1 may create a selective tumor-specific vaccination effect. We are currently investigating whether YIV-906 has the potential to promote vaccine effectiveness, such as in tumor vaccination.

We provided evidence supporting how YIV-906 could affect anti-PD1 activity to enhance the NFAT activity of Jurkat cells. Although the FDA has approved pembrolizumab and nivolumab as second-line monotherapies for the treatment of HCC in patients who have been previously treated with sorafenib, the complete response rate from those clinical trials is only about 3%–6% (El-Khoueiry et al., 2017; Finn et al., 2020). YIV-906 has great potential to increase the response rates and efficacy of these antibodies for HCC treatment. YIV-906 can also activate downstream cascades of TCR, which are the same cascades activated by most CAR constructs, and as predicted, YIV-906 can also promote NFAT activity of Jurkat cells expressing CAR-CD19 to enhance the killing power on tumor cells, independent of PD1-PD-L1 interaction. In this report we demonstrated that YIV-906 could promote two important types of immunotherapies currently in use: immune checkpoint antibodies and CAR T cell therapies. Since YIV-906 can modulate NFAT activity, which is critical for general T-cell activation, YIV-906 may also be combined with other treatments requiring T-cell activation.

Flavonoids such as baicalein, chrysin, and oroxylin A are key components of **S** that show enhanced NFAT activity. Flavonoids, with their various biological functions, are a major class of compounds found in many medicinal herbs used today (Panche et al., 2016). Thus, different flavonoids may have different immunomodulatory activities. In this study, we identified **S** (Scutelleria baicalensis Georgi) as the key herbal component responsible for NFAT modulation; this component ingredient has been reported to have an immune modulatory effect on Lewis lung tumor-bearing C57BL/6 mice by decreasing IL-17, IL-10, FOXP3, TGF-β1, RORγt, and IL-6 levels while remarkably increasing IL-2 and IFN-γ levels (Gong et al., 2015). Hesperetin and chrysin can enhance the activity of cytotoxic T lymphocytes *in vitro* (Sassi et al., 2017) or NK activity in BALB/c mice (Sassi et al., 2018). Baicalin-loaded poly (D,L-lactide-co-glycolide) (PLGA) nanoparticles can increase the infiltration of CD4^+^ or CD8^+^ T cells in tumors and induce a CTL cell response *in vivo* (Han et al., 2019). Further exploration of the potential use of flavonoid-containing herbs in the modulation of T cell function is worth considering. It should be noted that YIV-906s action is not identical to **S** or any of its compounds. Thus, the presence of other herbs or their compounds interaction plays a role in modulating the action of **S** in YIV-906.

**FIGURE 7:**
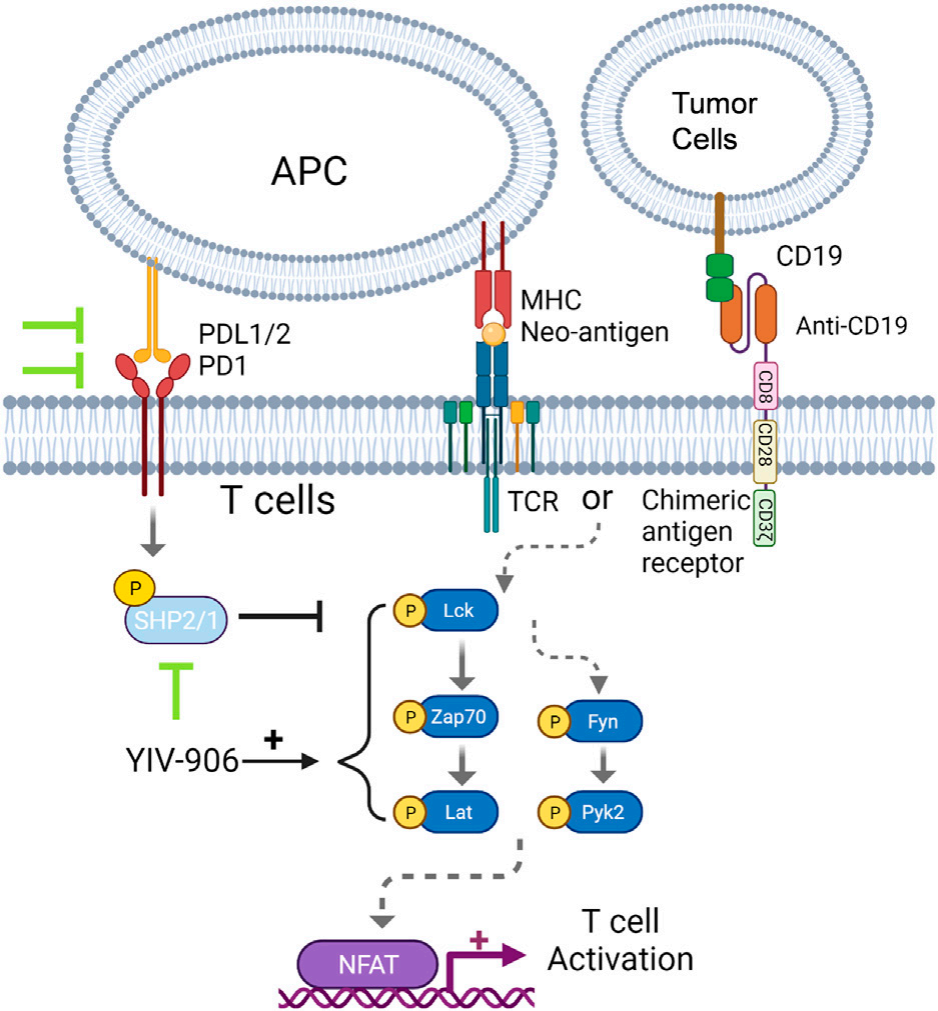
Schematic diagram for the mechanism action of YIV-906 for T cell activation.

In summary, YIV-906 can enhance the NFAT activity of effective T cells, partly by inhibiting SHP1/2 and inducing TCR downstream protein phosphorylation. **S** and its component compounds are involved in this process and may have different targets with different potencies within the tumor microenvironment. It is shown here that YIV-906 and perhaps some of its components could be used to modulate T cell activation for facilitating immune checkpoint blockade therapy or CAR T-cell therapy for the treatment of cancer patients Figure 7.

## Data Availability

The original contributions presented in the study are included in the article/Supplementary Material, further inquiries can be directed to the corresponding author.
