# Supplementary material for: YIV-906 enhances nuclear factor of activated T-cells (NFAT) activity of T cells and promotes immune checkpoint blockade antibody action and CAR T-cell activity
Source: Front Pharmacol. 2023 Jan 4;13:1095186. doi: 10.3389/fphar.2022.1095186 (PMC9846171; doi:10.3389/fphar.2022.1095186)
Supplement: Supplementary file 1 [file Presentation1.PPTX]

## Slide 1
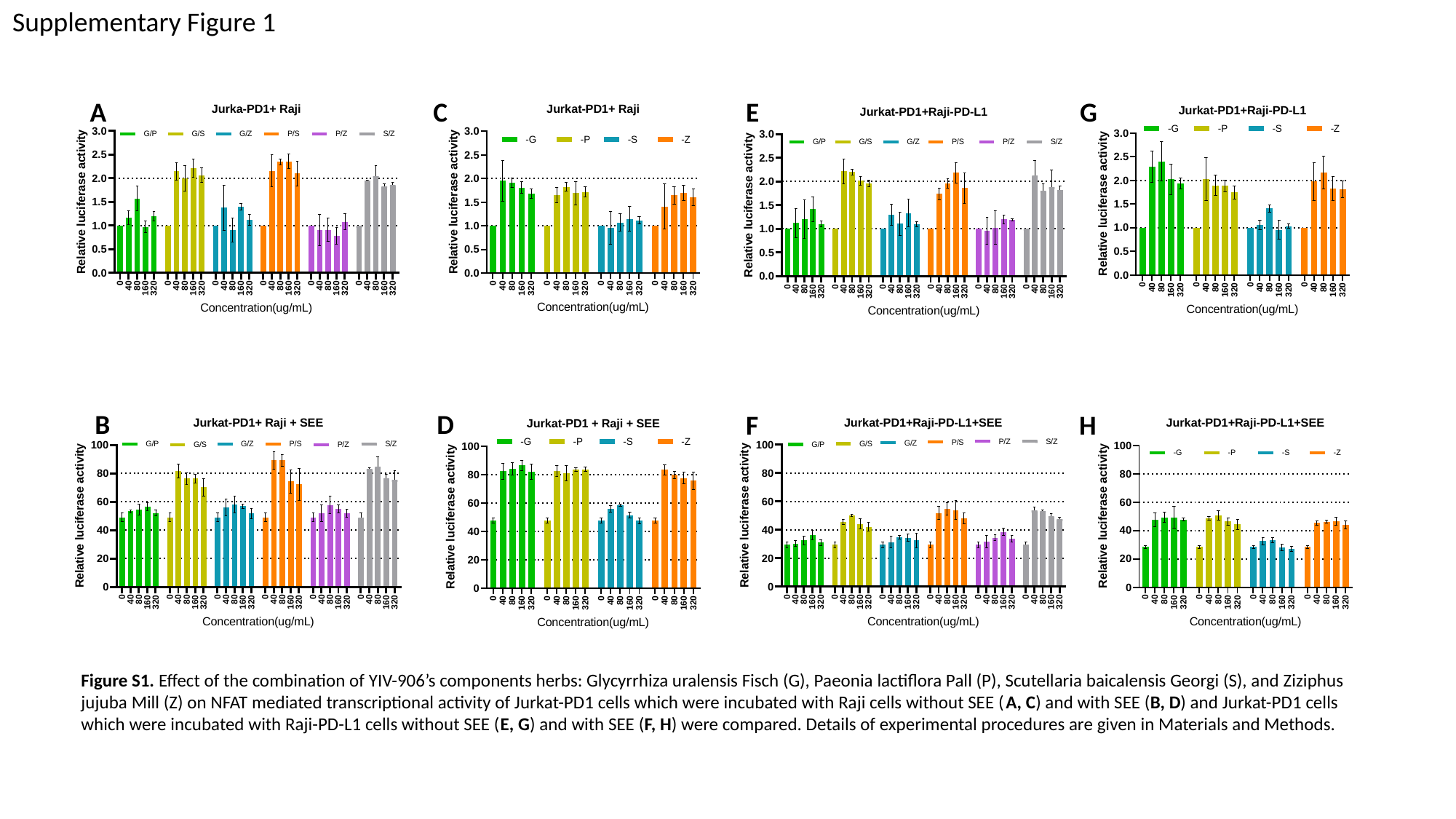

Supplementary Figure 1
A C
B D
E G
F H
Figure S1. Effect of the combination of YIV-906’s components herbs: Glycyrrhiza uralensis Fisch (G), Paeonia lactiflora Pall (P), Scutellaria baicalensis Georgi (S), and Ziziphus jujuba Mill (Z) on NFAT mediated transcriptional activity of Jurkat-PD1 cells which were incubated with Raji cells without SEE (A, C) and with SEE (B, D) and Jurkat-PD1 cells which were incubated with Raji-PD-L1 cells without SEE (E, G) and with SEE (F, H) were compared. Details of experimental procedures are given in Materials and Methods.

## Slide 2
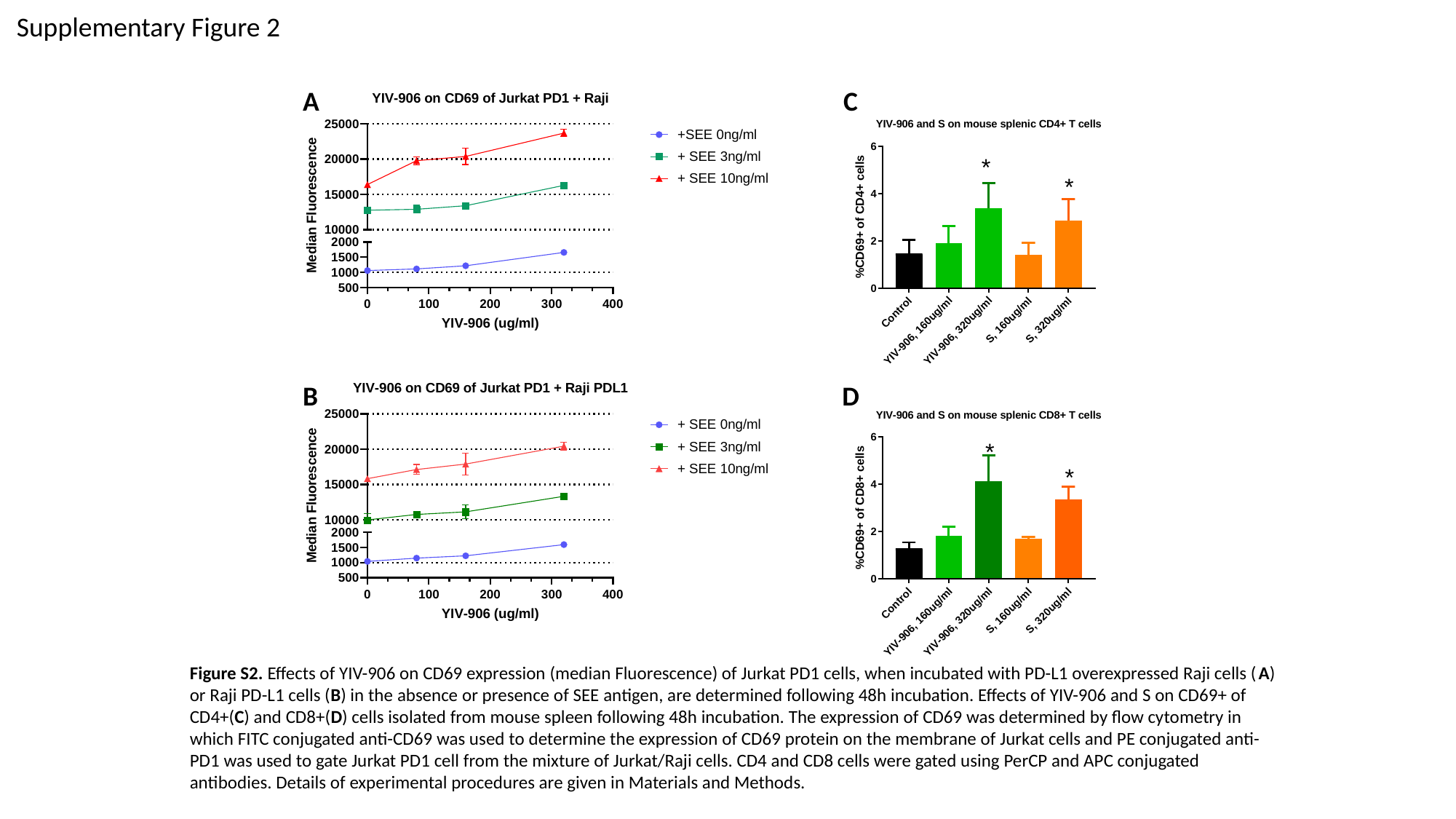

Supplementary Figure 2
A C
B D
Figure S2. Effects of YIV-906 on CD69 expression (median Fluorescence) of Jurkat PD1 cells, when incubated with PD-L1 overexpressed Raji cells (A) or Raji PD-L1 cells (B) in the absence or presence of SEE antigen, are determined following 48h incubation. Effects of YIV-906 and S on CD69+ of CD4+(C) and CD8+(D) cells isolated from mouse spleen following 48h incubation. The expression of CD69 was determined by flow cytometry in which FITC conjugated anti-CD69 was used to determine the expression of CD69 protein on the membrane of Jurkat cells and PE conjugated anti-PD1 was used to gate Jurkat PD1 cell from the mixture of Jurkat/Raji cells. CD4 and CD8 cells were gated using PerCP and APC conjugated antibodies. Details of experimental procedures are given in Materials and Methods.

## Slide 3
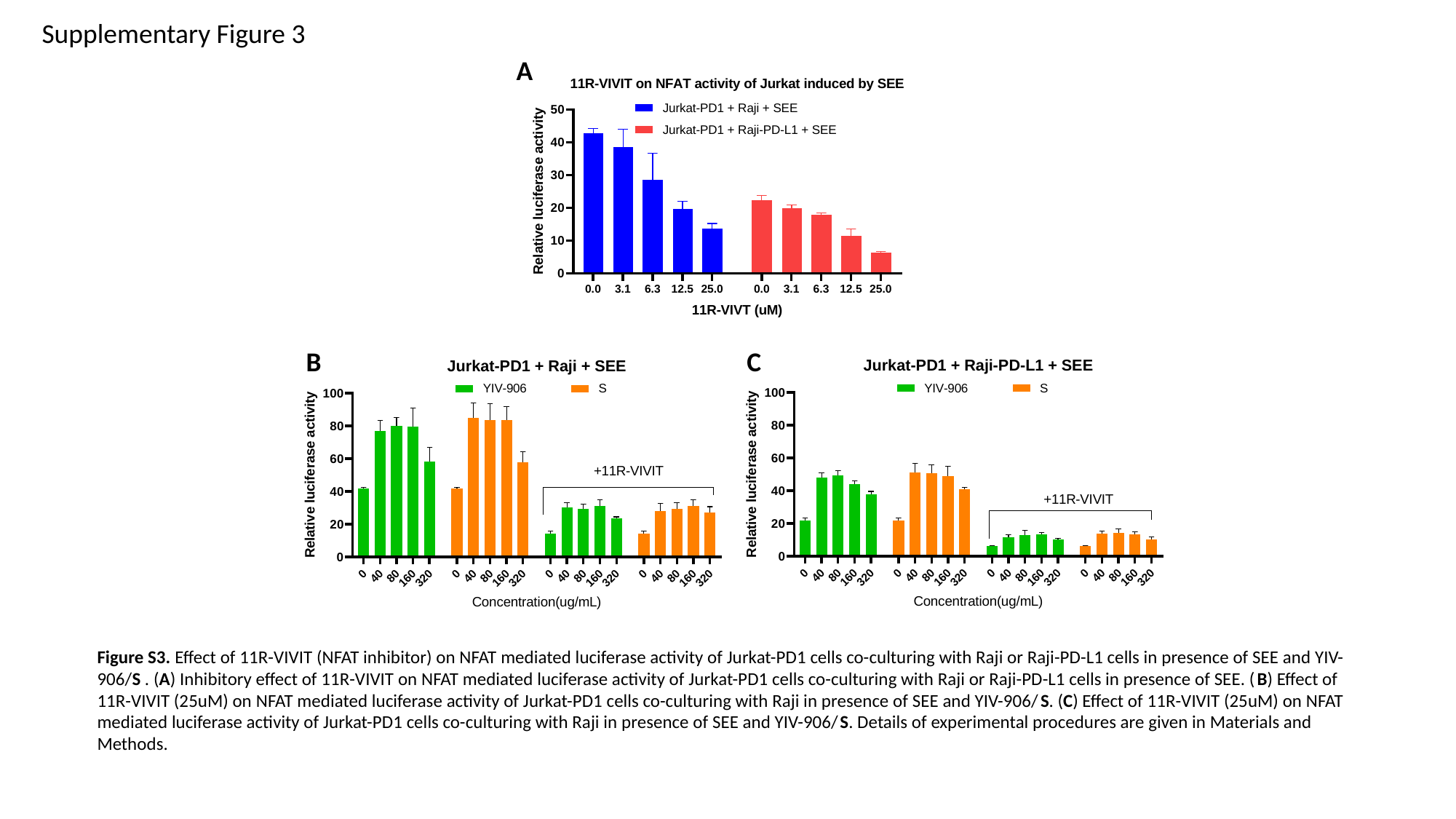

Supplementary Figure 3
A
B C
Figure S3. Effect of 11R-VIVIT (NFAT inhibitor) on NFAT mediated luciferase activity of Jurkat-PD1 cells co-culturing with Raji or Raji-PD-L1 cells in presence of SEE and YIV-906/S . (A) Inhibitory effect of 11R-VIVIT on NFAT mediated luciferase activity of Jurkat-PD1 cells co-culturing with Raji or Raji-PD-L1 cells in presence of SEE. (B) Effect of 11R-VIVIT (25uM) on NFAT mediated luciferase activity of Jurkat-PD1 cells co-culturing with Raji in presence of SEE and YIV-906/S. (C) Effect of 11R-VIVIT (25uM) on NFAT mediated luciferase activity of Jurkat-PD1 cells co-culturing with Raji in presence of SEE and YIV-906/S. Details of experimental procedures are given in Materials and Methods.

## Slide 4
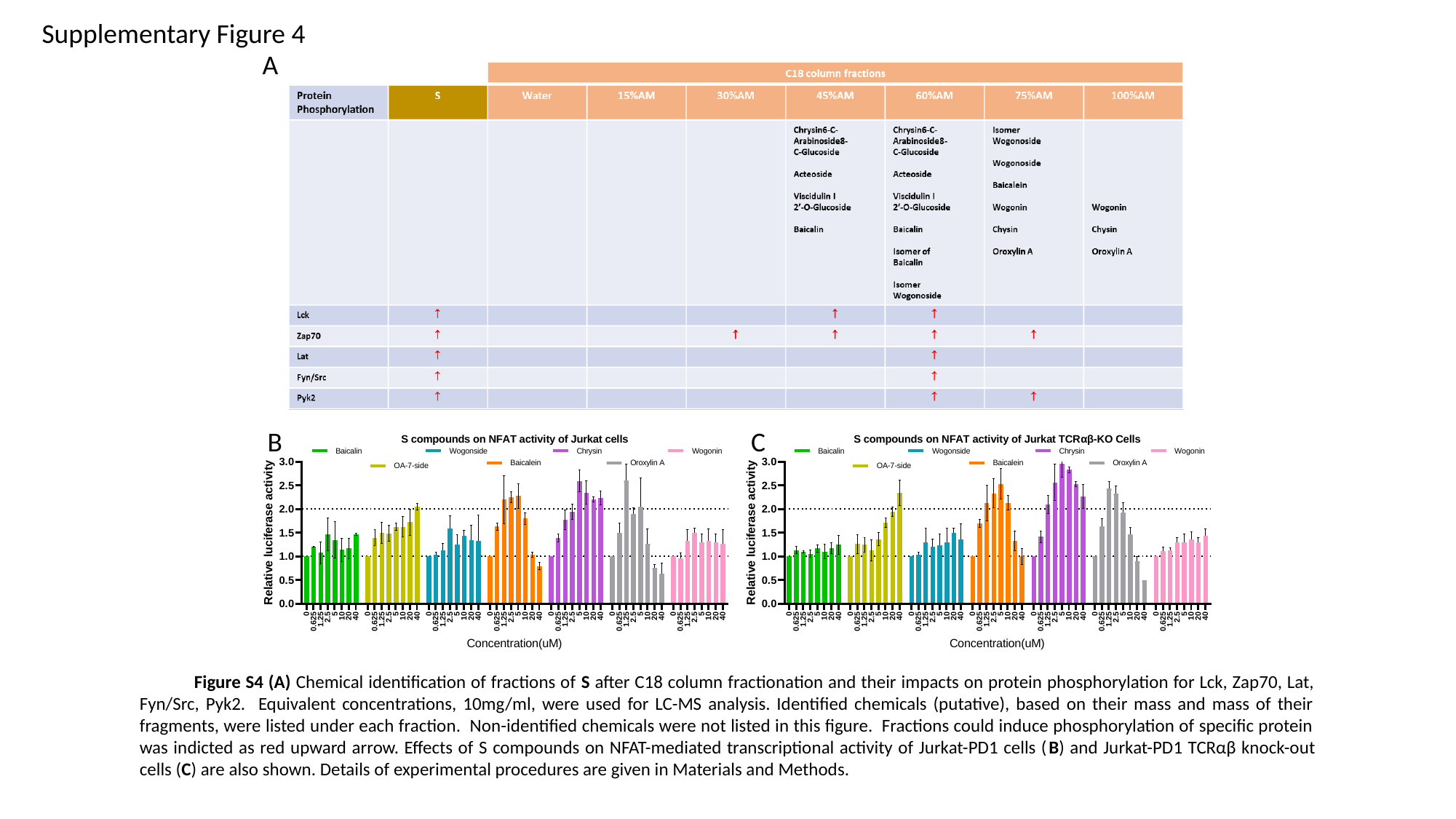

Supplementary Figure 4
A
B C
Figure S4 (A) Chemical identification of fractions of S after C18 column fractionation and their impacts on protein phosphorylation for Lck, Zap70, Lat, Fyn/Src, Pyk2. Equivalent concentrations, 10mg/ml, were used for LC-MS analysis. Identified chemicals (putative), based on their mass and mass of their fragments, were listed under each fraction. Non-identified chemicals were not listed in this figure. Fractions could induce phosphorylation of specific protein was indicted as red upward arrow. Effects of S compounds on NFAT-mediated transcriptional activity of Jurkat-PD1 cells (B) and Jurkat-PD1 TCRαβ knock-out cells (C) are also shown. Details of experimental procedures are given in Materials and Methods.

## Slide 5
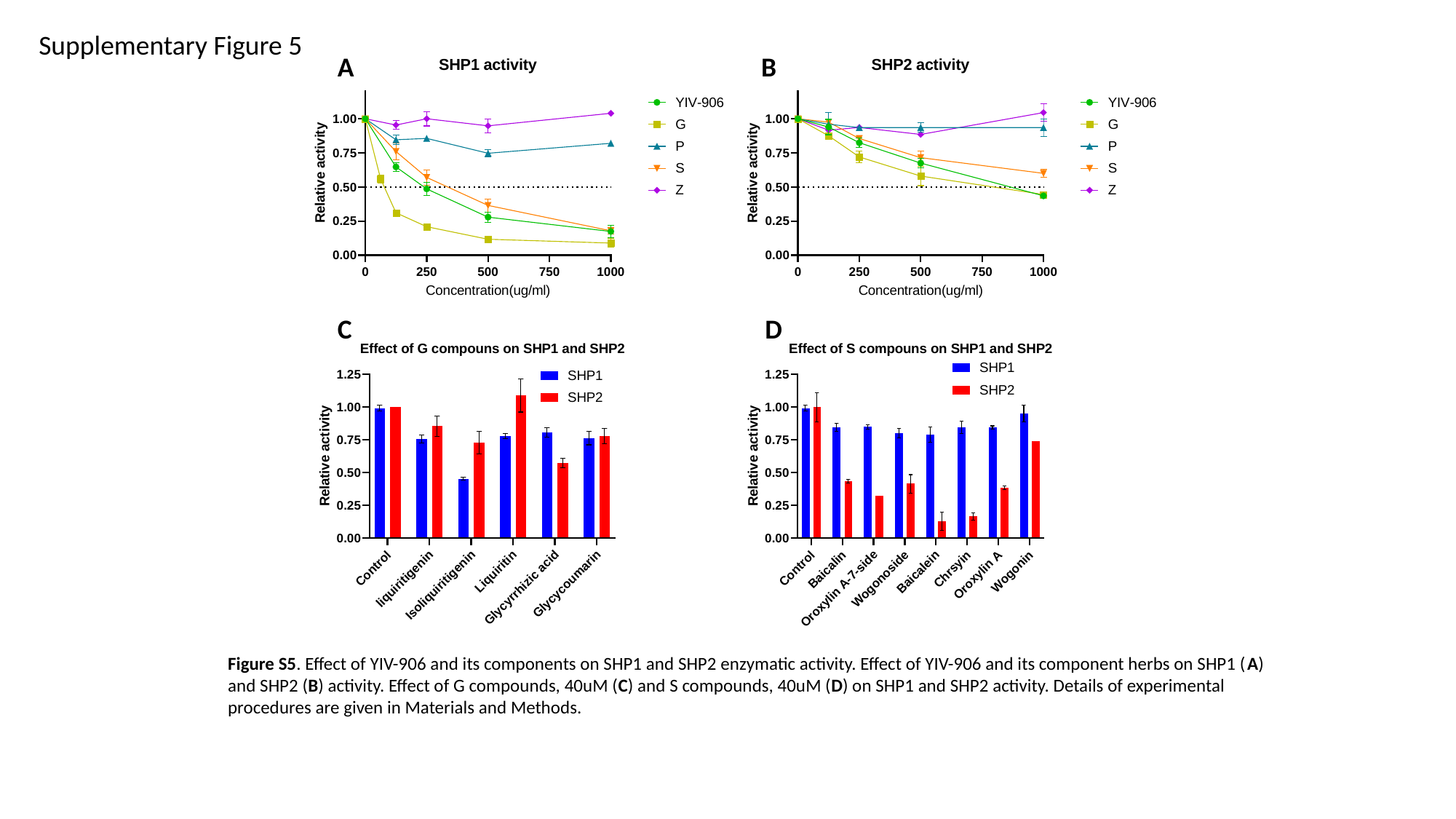

Supplementary Figure 5
A B
C D
Figure S5. Effect of YIV-906 and its components on SHP1 and SHP2 enzymatic activity. Effect of YIV-906 and its component herbs on SHP1 (A) and SHP2 (B) activity. Effect of G compounds, 40uM (C) and S compounds, 40uM (D) on SHP1 and SHP2 activity. Details of experimental procedures are given in Materials and Methods.

## Slide 6
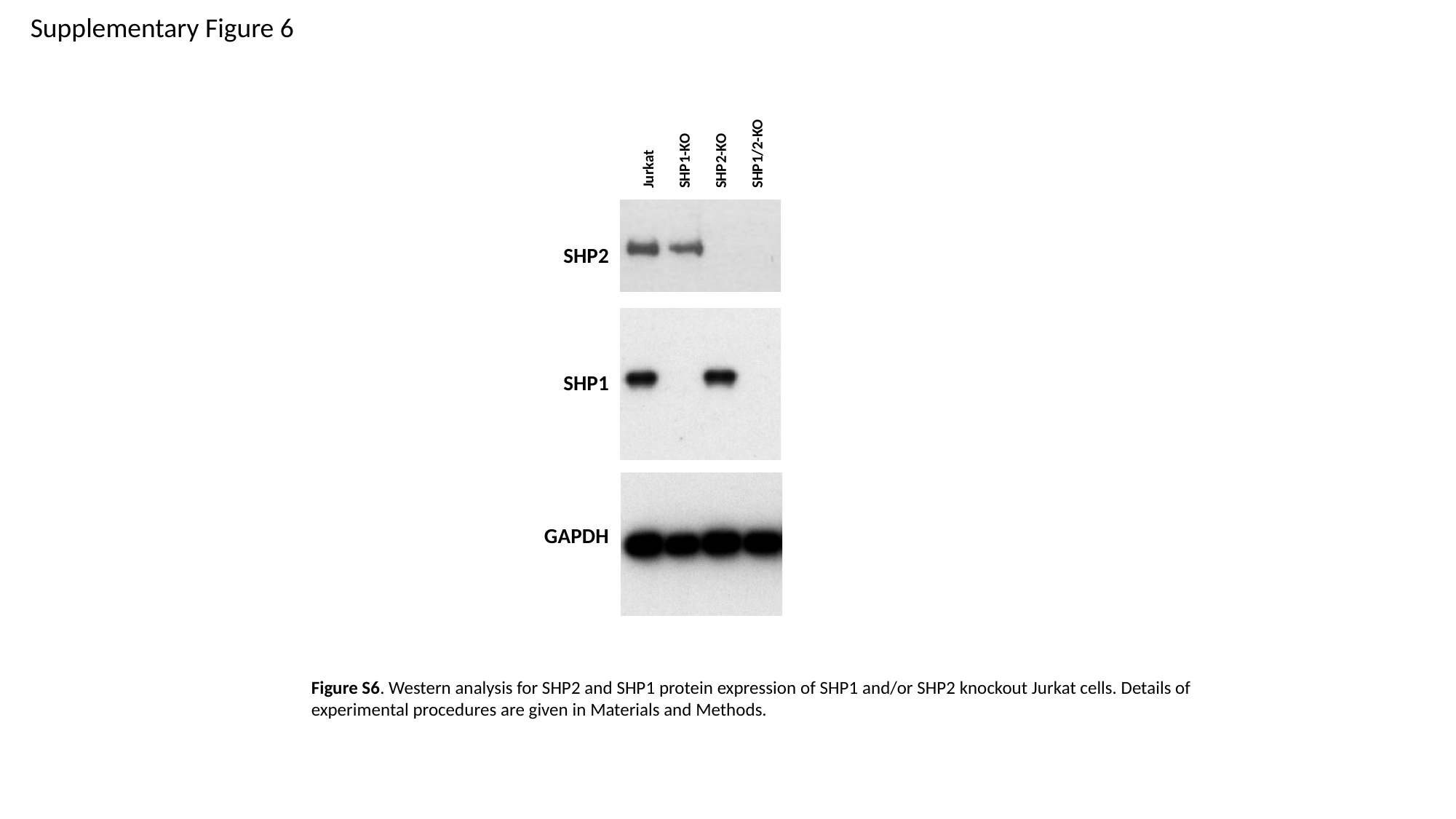

Supplementary Figure 6
Jurkat
SHP1-KO
SHP2-KO
SHP1/2-KO
SHP2
SHP1
GAPDH
Figure S6. Western analysis for SHP2 and SHP1 protein expression of SHP1 and/or SHP2 knockout Jurkat cells. Details of experimental procedures are given in Materials and Methods.

## Slide 7
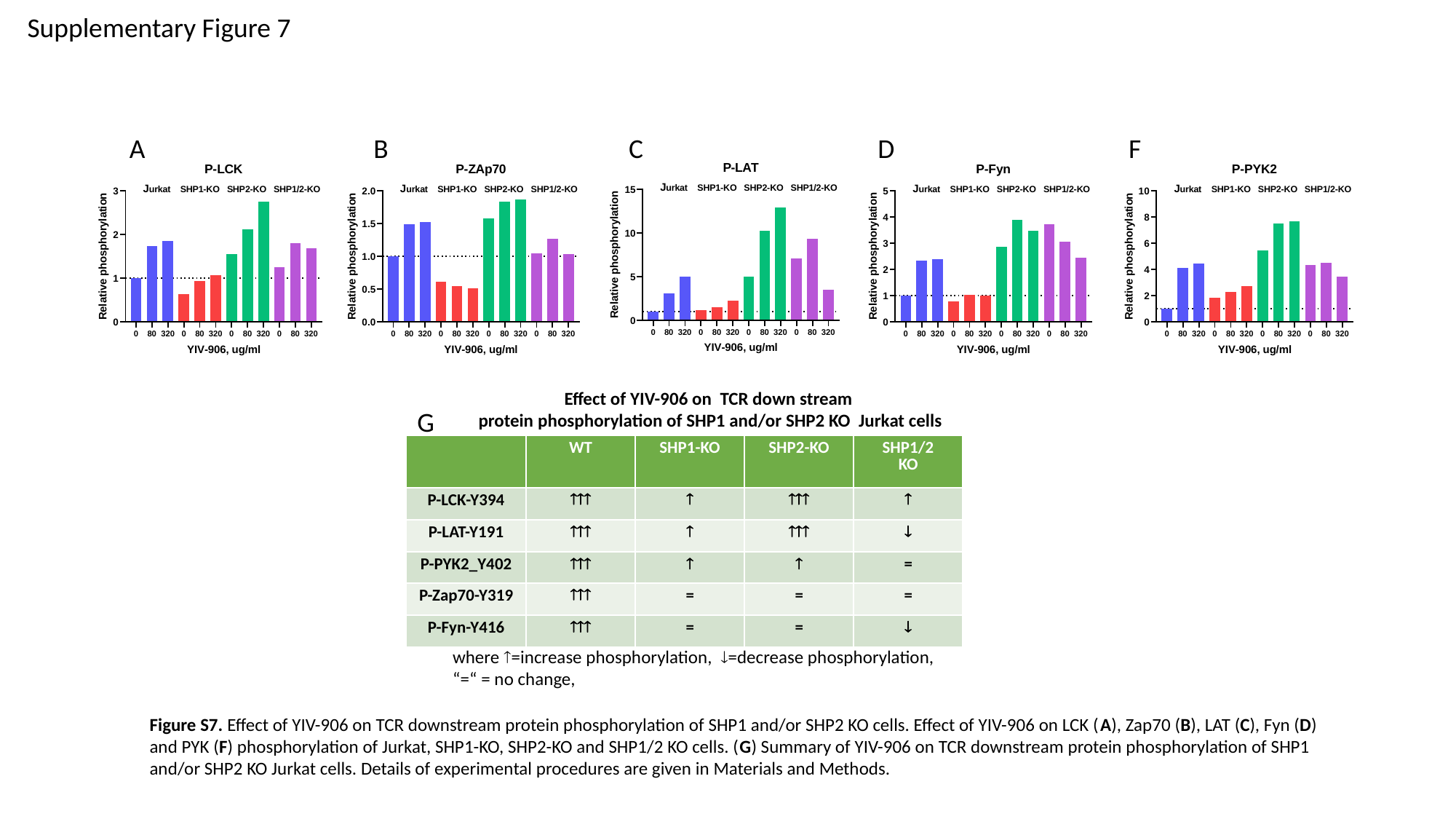

Supplementary Figure 7
A B C D F
Effect of YIV-906 on TCR down stream
protein phosphorylation of SHP1 and/or SHP2 KO Jurkat cells
G
| | WT | SHP1-KO | SHP2-KO | SHP1/2 KO |
| --- | --- | --- | --- | --- |
| P-LCK-Y394 |  |  |  |  |
| P-LAT-Y191 |  |  |  |  |
| P-PYK2\_Y402 |  |  |  | = |
| P-Zap70-Y319 |  | = | = | = |
| P-Fyn-Y416 |  | = | = |  |
where =increase phosphorylation, =decrease phosphorylation,
“=“ = no change,
Figure S7. Effect of YIV-906 on TCR downstream protein phosphorylation of SHP1 and/or SHP2 KO cells. Effect of YIV-906 on LCK (A), Zap70 (B), LAT (C), Fyn (D) and PYK (F) phosphorylation of Jurkat, SHP1-KO, SHP2-KO and SHP1/2 KO cells. (G) Summary of YIV-906 on TCR downstream protein phosphorylation of SHP1 and/or SHP2 KO Jurkat cells. Details of experimental procedures are given in Materials and Methods.

## Slide 8
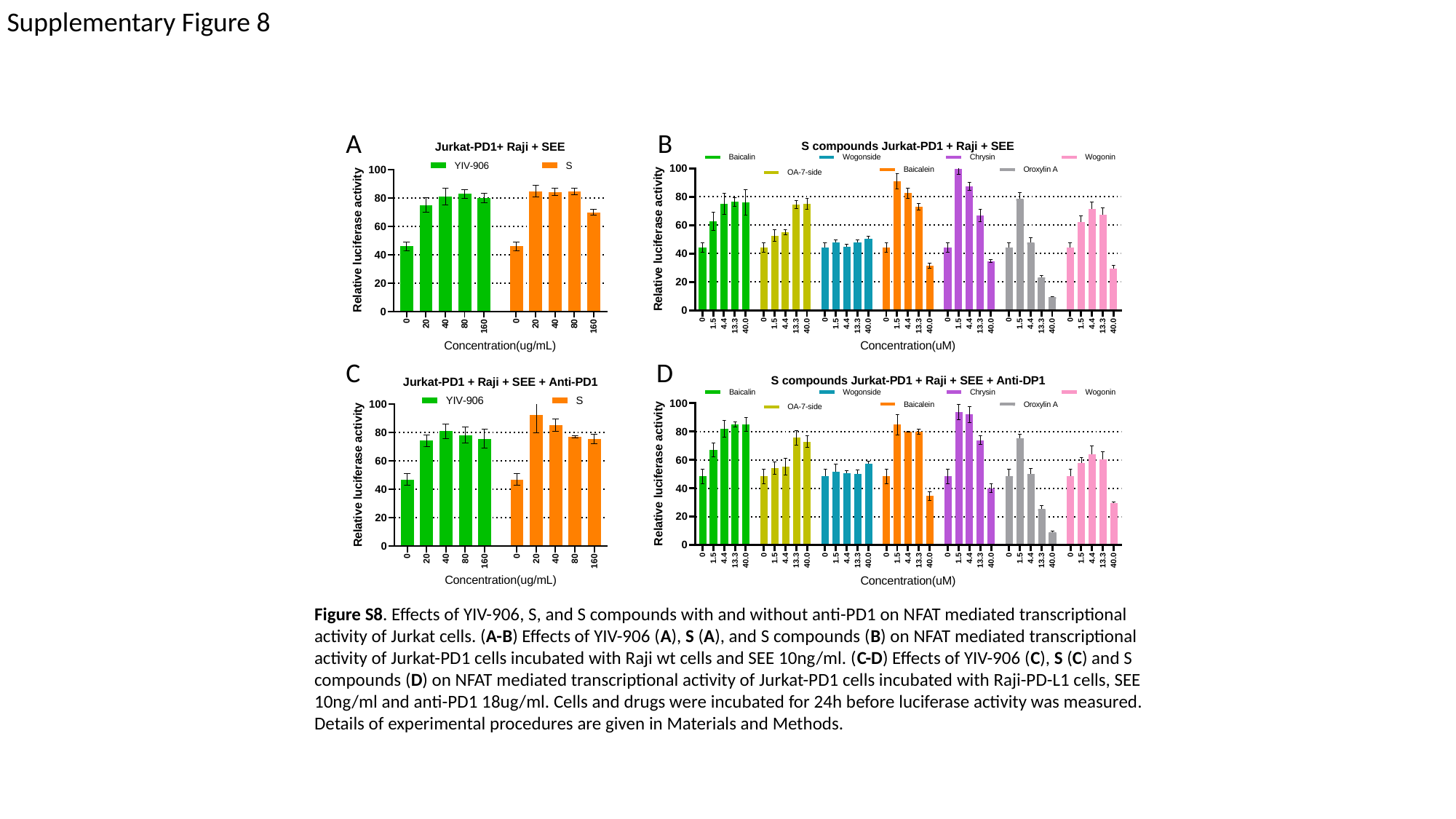

Supplementary Figure 8
A B
C D
Figure S8. Effects of YIV-906, S, and S compounds with and without anti-PD1 on NFAT mediated transcriptional activity of Jurkat cells. (A-B) Effects of YIV-906 (A), S (A), and S compounds (B) on NFAT mediated transcriptional activity of Jurkat-PD1 cells incubated with Raji wt cells and SEE 10ng/ml. (C-D) Effects of YIV-906 (C), S (C) and S compounds (D) on NFAT mediated transcriptional activity of Jurkat-PD1 cells incubated with Raji-PD-L1 cells, SEE 10ng/ml and anti-PD1 18ug/ml. Cells and drugs were incubated for 24h before luciferase activity was measured. Details of experimental procedures are given in Materials and Methods.

## Slide 9
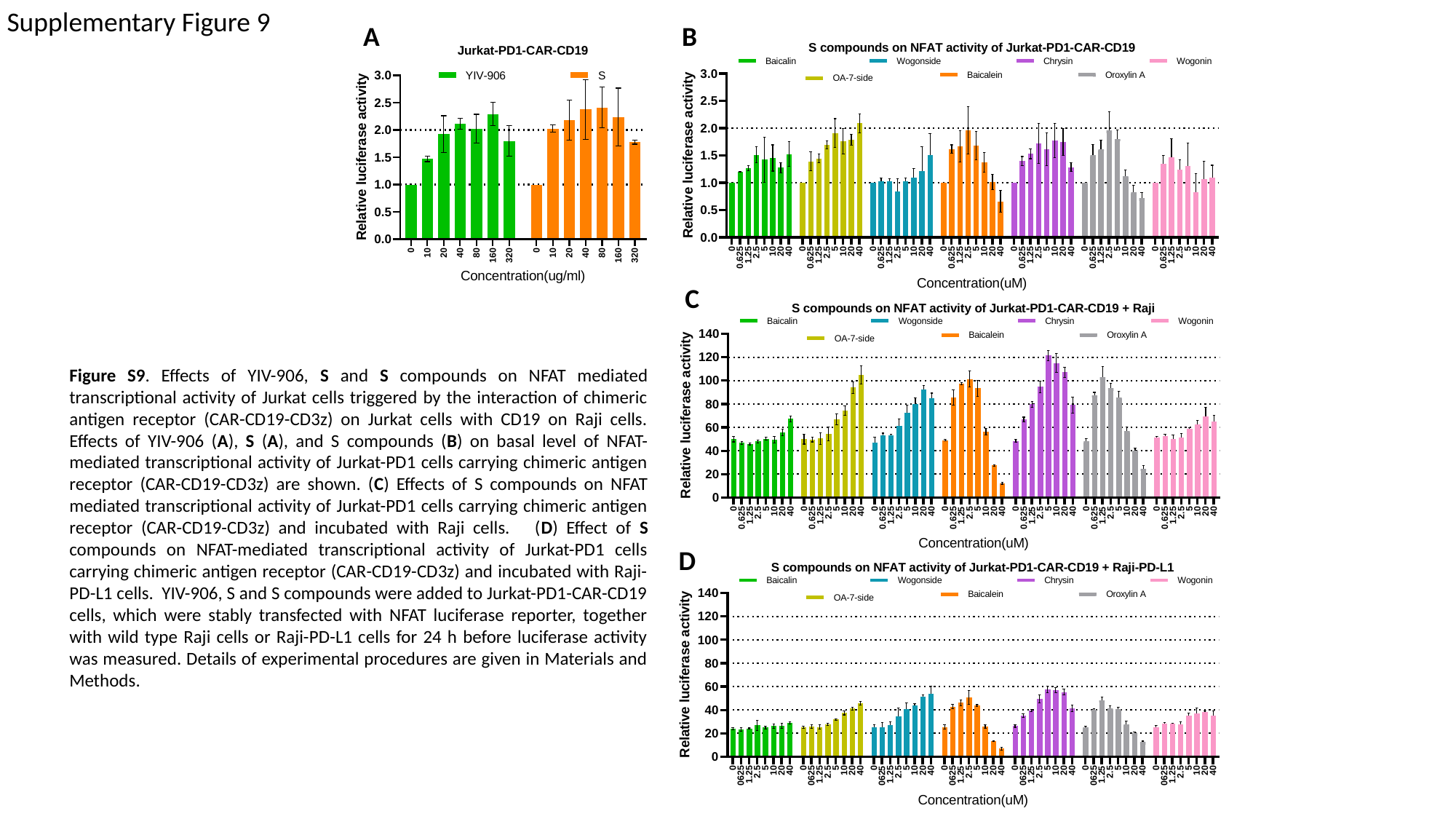

Supplementary Figure 9
A B
 C
 D
Figure S9. Effects of YIV-906, S and S compounds on NFAT mediated transcriptional activity of Jurkat cells triggered by the interaction of chimeric antigen receptor (CAR-CD19-CD3z) on Jurkat cells with CD19 on Raji cells. Effects of YIV-906 (A), S (A), and S compounds (B) on basal level of NFAT-mediated transcriptional activity of Jurkat-PD1 cells carrying chimeric antigen receptor (CAR-CD19-CD3z) are shown. (C) Effects of S compounds on NFAT mediated transcriptional activity of Jurkat-PD1 cells carrying chimeric antigen receptor (CAR-CD19-CD3z) and incubated with Raji cells. (D) Effect of S compounds on NFAT-mediated transcriptional activity of Jurkat-PD1 cells carrying chimeric antigen receptor (CAR-CD19-CD3z) and incubated with Raji-PD-L1 cells. YIV-906, S and S compounds were added to Jurkat-PD1-CAR-CD19 cells, which were stably transfected with NFAT luciferase reporter, together with wild type Raji cells or Raji-PD-L1 cells for 24 h before luciferase activity was measured. Details of experimental procedures are given in Materials and Methods.

## Slide 10
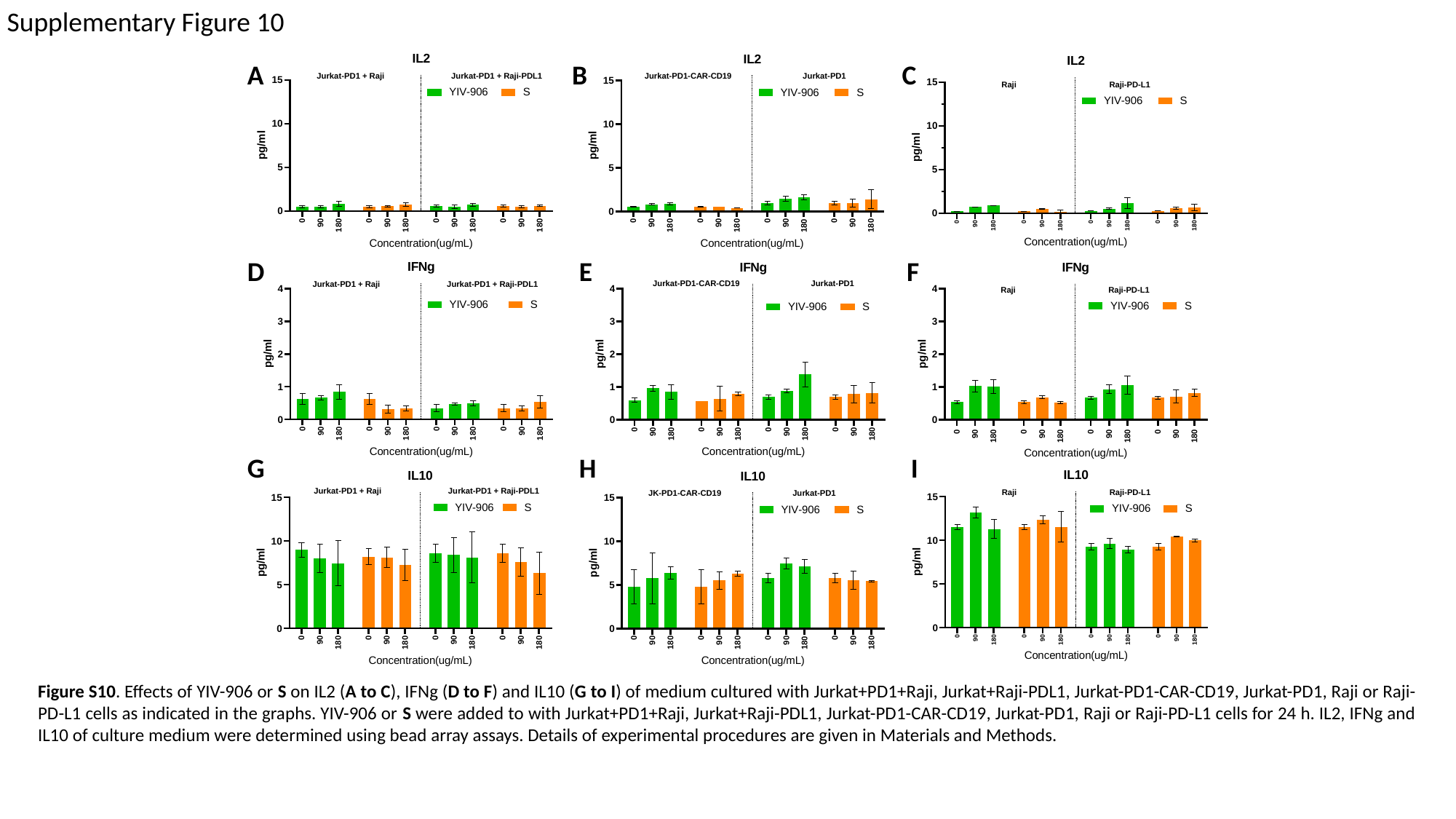

Supplementary Figure 10
A B C
D E F
G H I
Figure S10. Effects of YIV-906 or S on IL2 (A to C), IFNg (D to F) and IL10 (G to I) of medium cultured with Jurkat+PD1+Raji, Jurkat+Raji-PDL1, Jurkat-PD1-CAR-CD19, Jurkat-PD1, Raji or Raji-PD-L1 cells as indicated in the graphs. YIV-906 or S were added to with Jurkat+PD1+Raji, Jurkat+Raji-PDL1, Jurkat-PD1-CAR-CD19, Jurkat-PD1, Raji or Raji-PD-L1 cells for 24 h. IL2, IFNg and IL10 of culture medium were determined using bead array assays. Details of experimental procedures are given in Materials and Methods.

## Slide 11
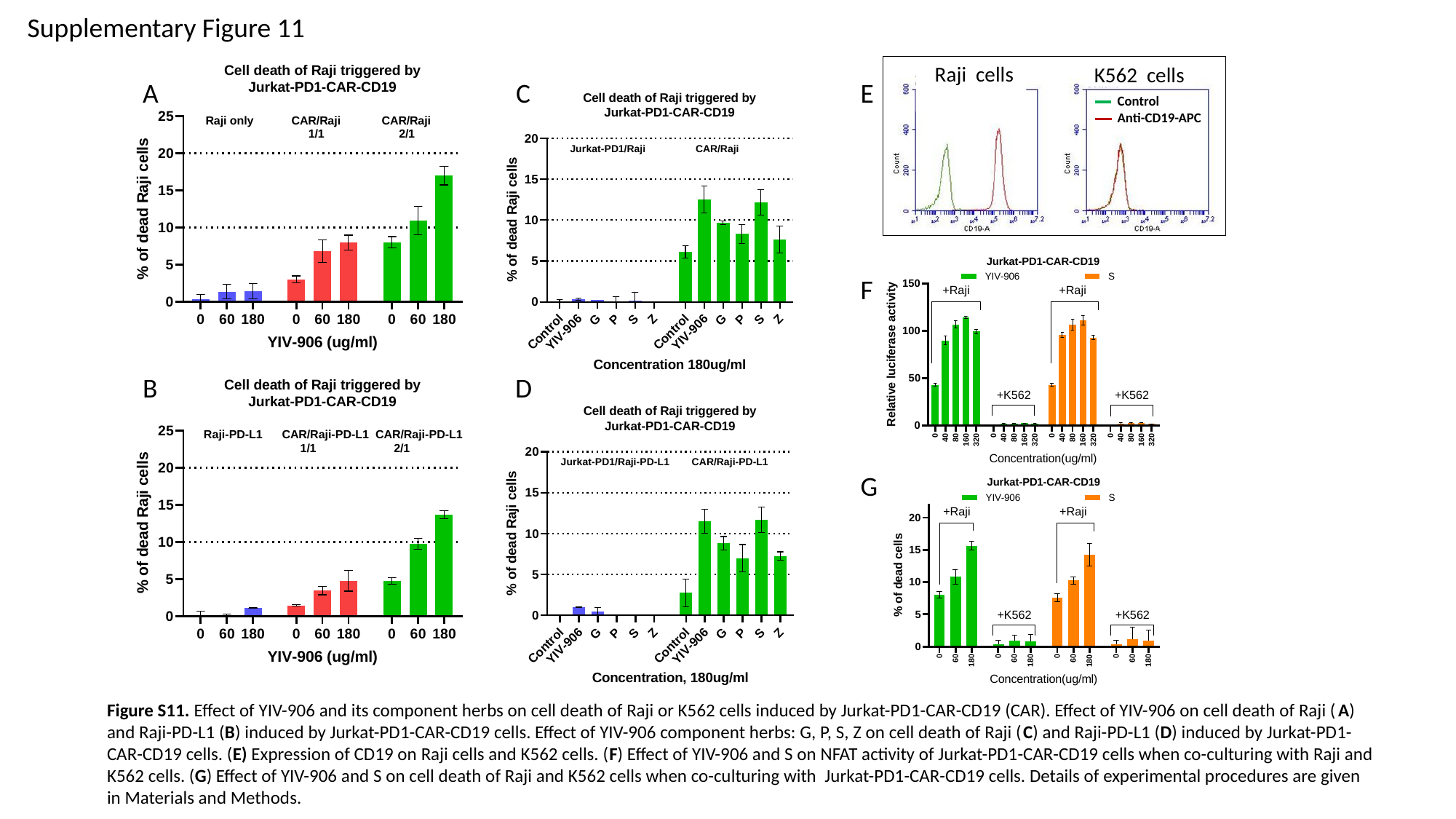

Supplementary Figure 11
 Raji cells
 K562 cells
Control
Anti-CD19-APC
A C
B D
E
F
G
Figure S11. Effect of YIV-906 and its component herbs on cell death of Raji or K562 cells induced by Jurkat-PD1-CAR-CD19 (CAR). Effect of YIV-906 on cell death of Raji (A) and Raji-PD-L1 (B) induced by Jurkat-PD1-CAR-CD19 cells. Effect of YIV-906 component herbs: G, P, S, Z on cell death of Raji (C) and Raji-PD-L1 (D) induced by Jurkat-PD1-CAR-CD19 cells. (E) Expression of CD19 on Raji cells and K562 cells. (F) Effect of YIV-906 and S on NFAT activity of Jurkat-PD1-CAR-CD19 cells when co-culturing with Raji and K562 cells. (G) Effect of YIV-906 and S on cell death of Raji and K562 cells when co-culturing with Jurkat-PD1-CAR-CD19 cells. Details of experimental procedures are given in Materials and Methods.

## Slide 12
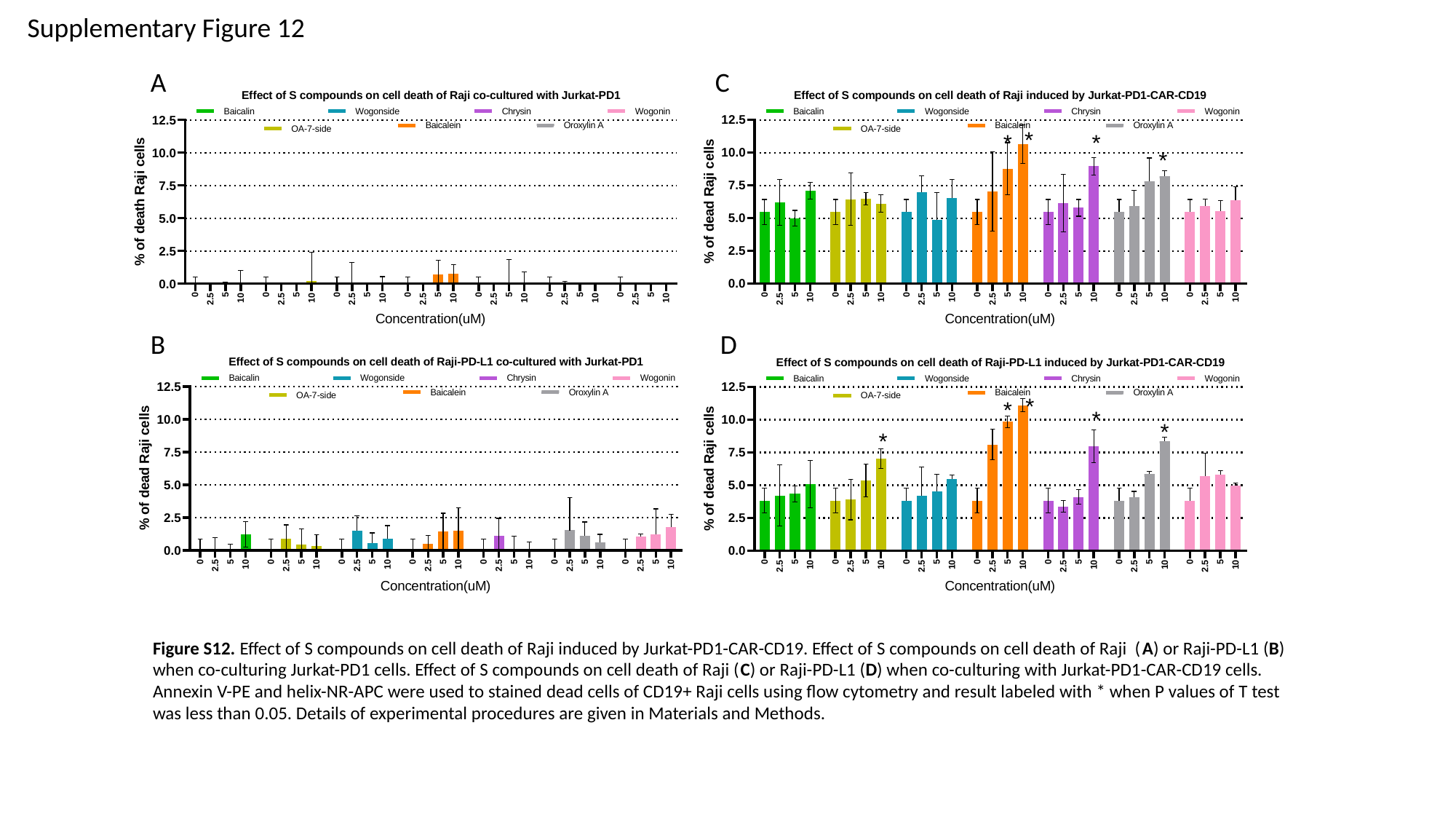

Supplementary Figure 12
A C
B D
Figure S12. Effect of S compounds on cell death of Raji induced by Jurkat-PD1-CAR-CD19. Effect of S compounds on cell death of Raji (A) or Raji-PD-L1 (B) when co-culturing Jurkat-PD1 cells. Effect of S compounds on cell death of Raji (C) or Raji-PD-L1 (D) when co-culturing with Jurkat-PD1-CAR-CD19 cells. Annexin V-PE and helix-NR-APC were used to stained dead cells of CD19+ Raji cells using flow cytometry and result labeled with * when P values of T test was less than 0.05. Details of experimental procedures are given in Materials and Methods.
